# Towards a practical use of text mining approaches in electrodiagnostic data

**DOI:** 10.1038/s41598-023-45758-0

**Published:** 2023-11-09

**Authors:** Roni Ramon-Gonen, Amir Dori, Shahar Shelly

**Affiliations:** 1 The Graduate School of Business Administration, Bar-Ilan University, Ramat Gan, Israel; 2 Department of Neurology, Sheba Medical Center, Tel HaShomer, Israel; 3 Sackler Faculty of Medicine, Tel Aviv University, Tel Aviv, Israel; 4 Department of Neurology, Rambam Health Care Campus, Haifa, Israel; 5 Neuroimmunology Laboratory, The Ruth & Bruce Rappaport Faculty of Medicine, Technion-Israel Institute of Technology, Haifa, Israel; 6 Department of Neurology, Mayo Clinic, Rochester, MN USA

**Keywords:** Peripheral nervous system, Health services, Public health, Biomedical engineering, Neurology, Neurological disorders

## Abstract

Healthcare professionals produce abounding textual data in their daily clinical practice. Text mining can yield valuable insights from unstructured data. Extracting insights from multiple information sources is a major challenge in computational medicine. In this study, our objective was to illustrate how combining text mining techniques with statistical methodologies can yield new insights and contribute to the development of neurological and neuromuscular-related health information. We demonstrate how to utilize and derive knowledge from medical text, identify patient groups with similar diagnostic attributes, and examine differences between groups using demographical data and past medical history (PMH). We conducted a retrospective study for all patients who underwent electrodiagnostic (EDX) evaluation in Israel's Sheba Medical Center between May 2016 and February 2022. The data extracted for each patient included demographic data, test results, and unstructured summary reports. We conducted several analyses, including topic modeling that targeted clinical impressions and topic analysis to reveal age- and sex-related differences. The use of suspected clinical condition text enriched the data and generated additional attributes used to find associations between patients' PMH and the emerging diagnosis topics. We identified 6096 abnormal EMG results, of which 58% (n = 3512) were males. Based on the latent Dirichlet allocation algorithm we identified 25 topics that represent different diagnoses. Sex-related differences emerged in 7 topics, 3 male-associated and 4 female-associated. Brachial plexopathy, myasthenia gravis, and NMJ Disorders showed statistically significant age and sex differences. We extracted keywords related to past medical history (n = 37) and tested them for association with the different topics. Several topics revealed a close association with past medical history, for example, length-dependent symmetric axonal polyneuropathy with diabetes mellitus (DM), length-dependent sensory polyneuropathy with chemotherapy treatments and DM, brachial plexopathy with motor vehicle accidents, myasthenia gravis and NMJ disorders with botulin treatments, and amyotrophic lateral sclerosis with swallowing difficulty. Summarizing visualizations were created to easily grasp the results and facilitate focusing on the main insights. In this study, we demonstrate the efficacy of utilizing advanced computational methods in a corpus of textual data to accelerate clinical research. Additionally, using these methods allows for generating clinical insights, which may aid in the development of a decision-making process in real-life clinical practice.

## Introduction

Text mining is a process that allows extracting previously unknown valuable knowledge from textual data. It enables eliciting implicit knowledge from unstructured texts and presenting it explicitly^1^. Over the past few decades, the amount of available daily medical information has been consistently growing, particularly data generated by healthcare professionals in their daily practice^2^. Uncovering knowledge hidden in this massive unstructured information is essential to supporting the daily decision-making process of medical professionals. In this context, text mining (TM) encompasses advanced techniques used to elicit high-quality structured information from unstructured textual data^3^.

The text mining process usually comprises several stages: (a) Preprocessing, where different natural language processing (NLP) techniques, such as tokenization and stemming, are used to standardize and clean unstructured textual input^4–6^; (b) Text representation, where the unstructured data is converted into a representation model such as Bag-Of-Words (BOW) or word embeddings^7^ that allows efficient analysis in subsequent phases^8^; (c) Discovery, where specific methods and techniques such as topic modeling, classification, and clustering allow extracting unexpected and unknown valuable information from textual data collections^9^.

Healthcare information systems gather massive amounts of textual and numeric information about patients, such as visits, prescriptions, physician notes, and more. Utilizing the textual content accompanying electronic clinical records can potentially improve healthcare quality, support clinical and research initiatives, decrease medical errors, and reduce costs. However, the variations in complexity, length, and technical vocabulary of health record documents challenge knowledge elicitation^10,11^.

EMR mining can potentially establish new patient-stratification principles and reveal unknown disease correlations^12^. Analyzing medical records is challenging, as they typically use unstructured plain texts and a specific technical vocabulary^13^. Moreover, physicians approach event or symptom descriptions differently, depending on their previous learning experiences and medical practices^14^.

In the present work, our goal was to demonstrate the utilization of large volumes of medical text to extract knowledge and generate meaningful insights in neuromuscular settings. We concentrate on identifying patient groups with similar diagnostic attributes using unsupervised topic modeling. We also examine differences between groups using demographic data and past medical history (PMH) derived from electrodiagnostic (EDX) evaluation reports. We demonstrate how such analysis can reveal hidden knowledge and generate new hypotheses.

### Text mining in neurology

Different researchers have used text-mining approaches in their medical studies, each according to the characteristics, guidelines, needs, way of action, and vocabulary of their field. Our focus was the rarely used text mining in neurology. Buchlak et al.^15^ reviewed 48 papers that use natural language processing (NLP) applications in clinical neurosciences. Although NLP and text mining are not identical, both aim to gain insights from unstructured data and share uses and techniques. The authors show that the NLP experience gained in the clinical neurosciences improved clinical data extraction, research literature synthesis, patient cohort identification, and clinical outcome prediction. A TM-based methodology developed by Kaya et al.^16^ allows the classification of predictable neuromuscular diseases concealed in the free medical history text of EMRs. They analyzed 150 PMH reports of five commonly encountered neuromuscular diseases—carpal tunnel syndrome (CTS), peripheral neuropathy (PNP), cervical radiculopathy (CRP), brachial plexus, and sciatic nerve injury. In their analysis, they compared a method they had developed, consisting of a keyword-based association algorithm with bi-grams, with the results of the Naïve Bayes algorithm. The comparison results showed an improved clinical decision support system (CDSS) in the pre-diagnostic period.

Diseases and syndromes from 65,000 neurology case reports were extracted from 66 PubMed journals^17^. They used TM methods for literature synthesis to explore the most frequent diseases and syndromes (DsSs), sort them into ten categories that reflect coinciding DsSs, and observe changes over time. They found that categories 3, 5, and 7 were most frequently discussed. The two most characteristic words of category 3 were dementia and atrophy, category 5—tremor and dystonia, and category 7—weakness and neuropathy.

Numerous examples exist of text mining benefits for various medical purposes, specifically clinical practice. Various researchers used NLP to retrospectively identify patients for inclusion in their study^18,19^. Karhade et al.^20^ developed an algorithm that processed clinical notes of spine surgery patients to identify those who needed reoperation for wound infection within 90 days of surgery. Other studies^24,25^ used NLP to extract data and glean new features from clinical texts. Several studies^26–28^ show the benefits of topic modeling for extracting relevant topics from patient support community websites and research articles, using latent Dirichlet allocation (LDA) modeling.

### Topic modeling of electronic health records

Topic modeling has been employed on clinical notes data for various objectives. On one hand, it serves the purpose of identifying clusters of patients who share common characteristics. This can aid in comprehending the extent of disease severity^29,30^, and identifying patients with comparable diagnoses, treatment plans, medication utilization, and related factors. Furthermore, it holds the potential to unveil novel International Classification of Diseases (ICD) codes and emergent trends^31^. On the other hand, for diverse predictive tasks, the extracted topics are used as features for classification algorithms^32–34^.

Topic modeling was used to uncover latent themes in clinical notes of patients with heart failure to help the clinical researchers infer the patient's severity of the disease, including related comorbidities. Before applying the topic modeling, a negation detection algorithm was used to create negated phrases. The topic modeling method was LDA to the tf–idf representation. To identify the theme for each topic, the representative notes that had a percent contribution of 0.80 or higher to the topic were used^29^.

The application of Bio Clinical Bert in conjunction with Bert extractive summarizer was introduced to shorten clinical data^35^. Furthermore, they implement topic modeling using LDA to assign relevant topics to the summary text. The MIMIC III dataset served as a source of clinical data. By that, they demonstrate they can shorten the text the clinician needs to read, eliminate noise, and extract knowledge from vast unstructured clinical data. Four temporal topic modeling techniques using clinical notes obtained from primary care electronic medical records were evaluated^36^. The evaluation methods were Nonnegative Matrix Factorization (NMF), Latent Dirichlet Allocation (LDA), Structural Topic Model (STM), and BERTopic. The study revealed that despite the heterogeneity in the statistical methodology of each model, the learned latent topical summarizations and their temporal evolution over the study period were consistently estimated.

Top2Vec was utilized to conduct topic modeling of Electronic Health Records (EHR) as a preliminary stage to generate features for a predictive model that was designed to identify individuals at higher fall risk. They demonstrate that the predictive performance of models that combine structured data with features derived from unstructured clinical notes outperformed those relying solely on structured data^33^.

The behavior of 17 topic modeling algorithms was studied using OCTIS and FuzzyTM packages and was evaluated based on their interpretability and predictive performance. The study didn’t identify a single model that outperforms the other models in both interpretability and predictive performance. In their experiments on a clinical notes dataset, the FLSA-W (fcm) performed best for interpretability, whereas ProdLDA seems the best choice for predictive performance^34^.

While topic modeling has found application in electronic health records and has gained traction in recent years, we have not come across text-mining or NLP papers that analyze nerve-conduction studies or textual EMG evaluation reports.

Neurological electrodiagnosis is an evolving field. Nerve conduction studies (NCS) and needle electromyography (EMG) are strong diagnostic tools used to evaluate patients with neuromuscular (NM) diseases. A verbal free-text extension attached to the electrodiagnostic (EDX) evaluation report describes the reason for referral and the EDX results and conclusions and summarizes the NCSs and needle EMG for the referring physician and the patient^39^. The report serves as a unique document that combines the results from various tests, combining machine-generated measurements with the neurologist's observations.

### Sex- and age-related differences in medicine

Few biological differences are associated with disease prevalence and incidence. Patient sex and age are the most important among them. Sex differences play a part in the etiology, manifestation, and treatment of disease^40^ and help understand the pathophysiological process and choose a treatment^41^. For example, females are more susceptible to multiple sclerosis and major depressive disorder than males and are at a greater risk of Alzheimer’s Disease in their lifetime. In contrast, males are more likely to be diagnosed with autism spectrum disorder, attention deficiency and hyperactivity disorder, and Parkinson’s Disease^5,6,40,42^.

Besides sex, age is another factor known to significantly impair nerve conduction, as age-related physiological changes in all organs and systems affect axonal health^43^. Some diseases tend to be age-specific, while others occur at all ages. One such example is myasthenia gravis (MG) known to be more prevalent in young females and older males, with a general higher incidence in females^44^.

### Examining the role of past medical history in the diagnostic process

A patient's past medical history (PMH) is a valuable diagnostic tool in medicine, as it can support or refute the need for further clinical laboratory or radiological testing. With the ongoing population growth, physicians must navigate a large volume of PMHs with detailed information about each condition. Therefore, simplifying the complexity of EMR-based decisions is crucial in many medical situations^45^. This study aims to determine the existence of associations between past medical history as phrased by the physician and unstructured diagnostic texts.

Previous research has explored the potential link between past medical conditions and future diagnoses. There are numerus examples in the literature linking medical history and diagnosis. One study^46^ that looked into the medical history of 73 patients (79% males) with brachial plexopathy found that falls were the primary cause of their injuries. Other examples concern diabetes mellitus (DM), one of the most serious twenty-first century global healthcare problems^47^. Diabetic neuropathies (DNs) are the most common complications associated with diabetes mellitus^48–50^. DN cases differ in their clinical course, distribution, fiber involvement (type and size), and pathophysiology, the most typical being a length-dependent distal symmetric polyneuropathy (DSP) with differing degrees of autonomic involvement^51^. Other studies investigated associations between DM and carpal tunnel syndrome (CTS)^52–55^ obtaining different results. Whether the source of heterogeneity in the research findings was due to differences in the target population or unmeasured confounding variables is yet to be assessed. A finer division of CTS patients into groups with specific characteristics might yield additional insights.

A common complication of breast cancer is brachial plexopathy, usually attributed to later effects of radiation^56^. Brachial plexopathy also emerges as a complication of motor vehicle accident (MVA) injuries^57,58^. Additionally, MVA's are a common cause of peripheral nerves injury (PNI)^57–60^.

**Figure 1:**
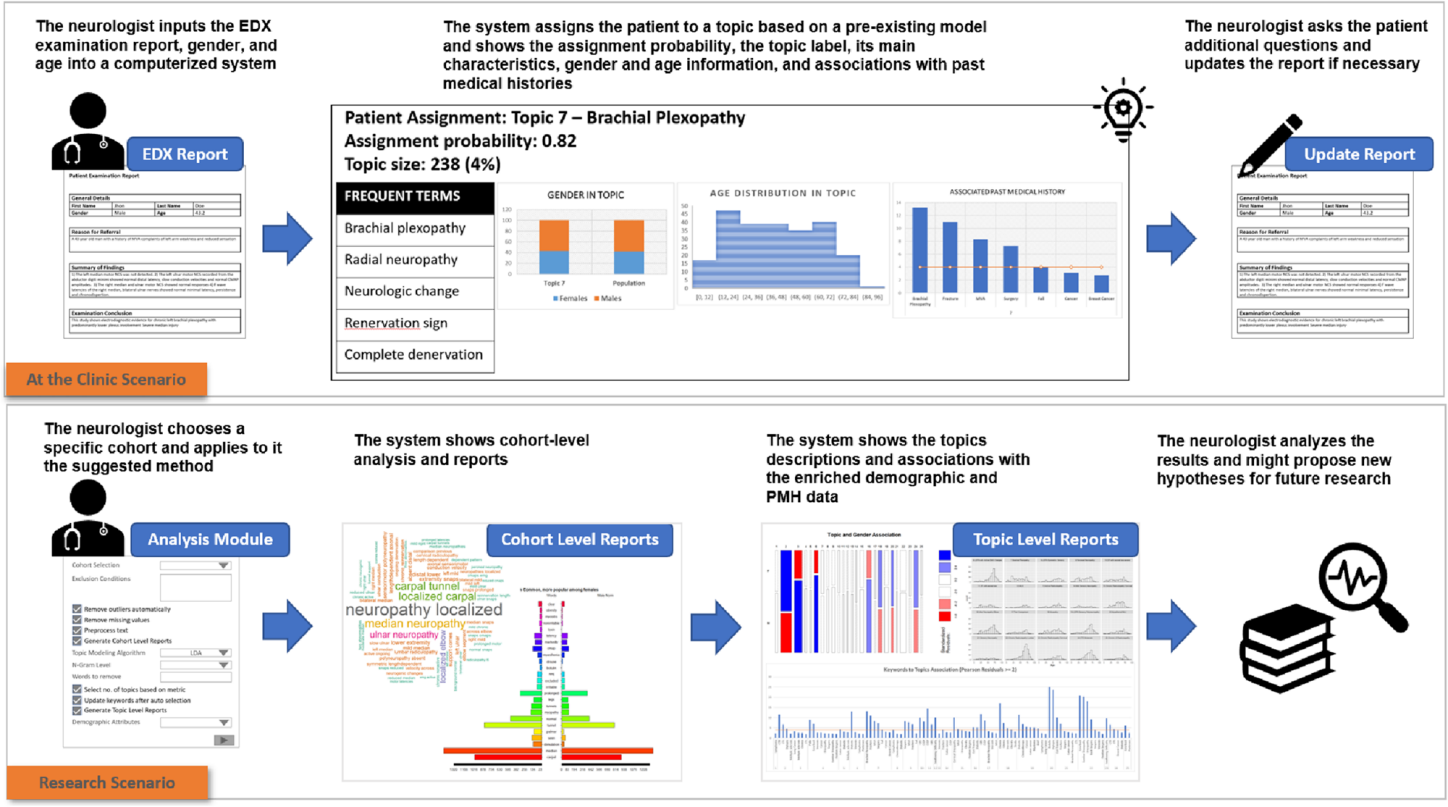
Flow diagram of the study’s rationale and practical use. The first scenario describes the contribution of our proposed framework at the point of care: The system assigns each patient visiting the clinic to a specific topic, shows the physician additional details on the topic and PMHs and proposes further recommended actions to the physician. The second scenario shows the advantages of the framework for research, knowledge discovery, and hypothesis generation.

In the present study, we show how text-mining approaches with computational techniques yield meaningful clinical insights. Our proposed method has several advantages: (a) Identifying and grouping patients with similar characteristics. (b) Analyzing age differences and showing that different topics each representing a group of patients with similar diagnostic characteristics have a different age distribution. (c) Detecting gender differences regarding various diagnoses. (d) Identifying associations between textual PMHs and diagnosis topics. The advantages listed above can aid in the development of healthcare analytics systems that will assist in generating research hypotheses. It can also assist in enhancing a clinical decision support system (CDSS) and developing an alert system that includes additional questions the physician might wish to ask the patient about past events and medical history. Figure 1 is a flow diagram of the study’s rationale and practical use.

## Methods

### Case collection

Sheba medical center (SMC) institution review board (9127-22-SMC) approved the study before its launch. The electronic medical record system at Sheba Medical Center served to identify patients who underwent comprehensive EMG/NCS tests between May 2016 and February 2022.

Data retrieved from multiple data clouds were assembled to form one data warehouse. The resultant dataset included records from hospitalizations and outpatient clinic visits. Besides demographic variables such as patient age and sex, the data for each visit comprised the visit date and textual medical notes stating the reason for referral, a summary of findings, and the physician's test readout conclusions. Data preparation consisted of filtering to remove incomplete records and a primary text analysis to identify tests with normal/abnormal results. A record was defined as "normal" if the performing electromyographer's report stated that the NCS/EMG results were within the normal range. The present study engages only with the abnormal test population.

### Inclusion criteria

We included patients who underwent a detailed electrophysiological evaluation after a neuromuscular specialist saw them in our EMG laboratory. Patients whose data (age, sex, or medical notes) were incomplete were excluded. Multiple tests of the same patient on different dates were included in the topic modeling stage (as the summary notes for each visit were not the same). Only a patient's last visit participated in the statistical analysis to create independent records.

### Text preprocessing

Of the different textual medical notes' sections, we focused on the conclusions, which contain the main findings of the nerve conduction study (NCS) and EMG results and the reason for referral that comprises a brief medical history, and the tested medical condition. A text preprocessing stage performed to "clean" the text and prepare it for analysis, included lowercasing the text, deletion of stop words and a few words commonly used in this specific domain, and punctuation marks. Only numbers that were parts of letter-number combinations were kept. (e.g., 1. Was removed but c67 was not). The default function that creates the term document matrix deletes words of length one or two, we changed these settings and kept length 2 terms (e.g., DM, MM). Lemmatization was performed in the topic modeling stage with the LDA configuration. We benefited from the advantage that most reports had a similar structure. This allowed us to identify different sections (e.g., reason for referral, conclusion), and define several regular-expression rules to identify past medical history and negation (diagnoses the examination did not confirm and therefore were deleted. For example, “there is no evidence of”).

### PMH keywords/terminology extraction

Keywords represent the central themes of a text collection whose volume is too large to analyze manually. Keyword extraction stands for an automatic identification of key terms^61,62^. In this study, we used keyword extraction techniques to enrich the data by creating additional features representing patients' past medical history.

The first stage of identifying keywords clinicians use to describe the past medical history of patients involved extracting texts appearing after the words “history of” in the reason for referral section. Although not all clinicians use the term “history of”, it was quite common, appearing in 3447 (56%) of the visit reports. For this purpose, we constructed a new dataset containing the text that starts with “history of" and ends with the string “complain”, which was the standard writing pattern, and promised to contain the keywords we were seeking. Another option, more generic, is to search the end of the sentence. Creating this smaller dataset allowed us to keep only terms in the context of past medical history. This dataset was used only for the keyword extraction stage. To reduce the number of terms in the dataset we removed sparse terms and kept only terms that appeared at least in ten documents. This step significantly reduced the number of terms. The standard preprocessing stages were performed to clean the data and normalize the terms (lowercasing, stopwords, numbers and punctuation removal and lemmatization), in addition we created several rules to normalize similar concepts. For example, bone marrow transplant can be written also as ‘bmt’, ‘bm transplantation’ and ‘bone marrow transplantation’. Normalizing concepts is important to find ‘not obvious’ associations because we might reveal an association or make it statistically significant only if we have enough support in the data by uniting terms that convey the same meaning. The list of normalized concepts appears in appendix G. To create the list of normalized terms, we examined the frequent abbreviations and looked for the partial terms of their long format, for example we searched for the word ‘marrow’ and examined the words next to it. Several abbreviations can have different meanings, for example DM can stand for diabetes mellitus but also for dermatomyositis, to make sure we use the correct concept we consulted a neurologist and checked the full form of the abbreviation is a frequent term in the dataset. We also looked at the Meta-inventory database^63^ that provides a list of medical terms, their abbreviations, acronyms and similar concepts.

After evaluating several keyword extraction methods, we chose to use for this work one of the most dominant statistical approaches^61^, term frequency-inverse document frequency (TF-IDF)^64^, along with a bigram frequency analysis. TF-IDF (Eq. 1) calculates a score for each term in each document considering both the term's frequency in the document and how common (or uncommon) a term is amongst the corpus. This method discerns both frequent and rare terms in the corpus. After calculating the score per term and document we summed a total score for each term.

1 $$\begin{aligned} & TFIDF_{score} \left( {t,d,D} \right) = TF\left( {t,d} \right)*IDF\left( {t,D} \right) where\,t\,is\,a\,term\,and\,d\,is\,a\,document\,in\,corpus\,D \\ & TF = \frac{term\,t\,occurences\,in\,document\,d}{{total\,terms\,in\,the\,document\,d}}, \quad IDF = {\text{log}}\left( { \frac{total\,documents\,in\,corpus\,D}{{number\,of\,documents\,with\,term\,t\,in\,it}}} \right) \\ \end{aligned}$$

Equation 1: TFIDF score calculation for term t in document d.

After listing the keywords with their TF-IDF weight and frequent bigram list, a neurologist went over the lists and refined them, keeping words related to a medical history.

After extracting the keyword list, we returned to our full cohort dataset and searched for each keyword in the reason for referral section. Once keywords were identified, we used them to create new features indicating a patient's past condition. This resulted in a dataset that contained the patient ID and features that indicate whether he had a specific past medical history or not.

### Topic modelling

The primary purpose of topic modeling is to identify latent topics in a set of documents. Over the years, numerous topic modeling algorithms have been developed. Latent Dirichlet Allocation (LDA) stands out as the most widely used algorithm for topic modelling based on statistical analysis^65^ and has been used in most healthcare applications^66–68^. LDA is an unsupervised, probabilistic method built upon the concept that documents can be modeled as a mixture of latent topics, where each topic is a distribution over words^69^. There are multiple variations of LDA, one of the widely used is LDA with Gibbs sampling^70^, which is a Markov Chain Monte Carlo (MCMC) technique used for approximate inference in probabilistic models and is effective in handling high dimensional probabilistic models such as LDA^71–73^. However, LDA has several reported limitations. These include the necessity for data cleaning and pre-processing, the selection of model parameters, such as the number of topics, and challenges related to the interpretability and validation of the generated topics^74–76^. To tackle these limitations, in recent years, more advanced algorithms have been invented as an alternative technique for topic modeling, such as cluster analysis of document embeddings. Two notable advanced neural models techniques^77,78^ that offer automated pipelines for neural topic modeling are Top2Vec^76^ and BERTopic^79^. In their general pipelines, both methods begin with a collection of input texts. They then create document embeddings, followed by reducing the size of the embedding vectors through a dimensionality reduction algorithm. Subsequently, HDBSCAN is applied to cluster the reduced vectors and at the end they generate topic representations. There are two key distinctions between these two methods, the first is that BERTopic generates document embedding using pre-trained transformer-based language models. In contrast, Top2Vec, in addition to pre-trained embeddings, provides the option to train a doc2vec embedding model specifically on the given corpus. A second distinction arises in the way each algorithm generates the terms that characterize individual topics. BERTopic employs a class-based TF-IDF (C-TF-IDF) procedure while Top2Vec calculates the similarity of each term to the cluster centroid. Both algorithms automatically detect the number of topics and have an option to reduce the number of topics based on similarity between the generated topics.

When conducting LDA, the algorithm does not automatically detect the number of topics, and over the years, several evaluation measures have been developed to assist in determining the appropriate number of topics. External evaluation measures are applied when a ground truth exists, and the number of topics or classes is known beforehand. Internal measures, on the other hand, come into play when no ground truth is available. Among the most commonly used internal evaluation measure for topic modeling is the perplexity of a held-out dataset with respect to an inferred model^69,80,81^. This measures a model’s ability to generalize and predict newly-presented documents, and is based on the model’s likelihood^82^. Lower perplexity values indicate improved model performance. Moreover, the ldatuning R library^83^ provides four indices to estimate the optimal number of topics, one of these indices is Griffiths2004, a prominent evaluation measure that computes the log likelihood (Log(P(W|T))^84^. A higher log likelihood indicates a better fit of the model to the data. The log likelihood measures how well a topic model fits the observed data, while perplexity, measures how well the model generalizes to unseen data. Additionally, a manual evaluation, which still remains critical^80^, was also carried out.

In our study we examined the python packages BERTopic (version 0.15.0) using its default hyperparameters, Top2Vec (version 1.0.29) with both doc2vec embeddings and pre-trained universal-sentence-encoder embeddings. Additionally, we examined Gibbs-LDA from the R topicmodels package (0.2–14), with unigrams, bigrams and their combination. The inclusion of bigrams in the analysis introduces an extra semantic layer. For Gibbs LDA we conducted five-fold cross validation and computed the perplexity on each held-out set to evaluate a range for the number of topics spanning from 2 to 100. This process parallels the approach described in^85,86^. Subsequently, once the range was established, we used the Griffiths2004 measure in conjunction with a manual evaluation to ascertain the definitive number of topics.

To our knowledge, there are no papers that have previously employed topic modeling for Nerve Conduction Studies and EMG reports. In our research, we consider the conclusion text of the EDX reports as individual documents and employ topic modeling to form groups of patients with some similarities in clinical impressions and diagnoses.

### Visualizations

Data visualization plays an increasingly important role in analytical processes, complementing the statistical and data mining methods. It ranges from basic charts and graphs that elucidate trends, patterns, and outliers, to executive dashboards and guided analytics^87^. Visualizations intended to simplify the representation of a large amount of information play a central role in healthcare information systems.

We used several visualization types to demonstrate prominent text characteristics in our extensive text collection. A normalized pyramid plot shows differences in the number of each word's occurrence among males or females. Because the number of male visits is higher, we calculated a proportional value. Furthermore, to validate the outcomes of the frequency analysis, we performed the Chi-Square test to ascertain the statistical significance of the observed variations.

Two visualizations show topic and PMHs associations. In the first one, a topic-keyword association plot, a two-level X-axis displays topics and the keywords associated with them. A keyword appears under a topic if the standardized Pearson residuals (marked R) are greater than or equal to 2 (association indication). The Pearson residual score sorts out the keywords. A score higher than 4 indicates a strong association. At this point we added a target line, emphasizing all associations above 4. The second visualization, the keyword-topics association plot, provides another view of the same data, showing the topics associated with each keyword, each bar showing the associated topics. The length of a bar represents the strength of the association relative to other topics in the same bar. For example, if keyword 1 is associated with two topics, in one the R = 5 and in the other R = 10, 33.3% of the bar will show the color of topic 1 and 66.6% the color of topic 2. The visualizations were generated using both R and Excel.

### Statistical analysis

To avoid dependent observations in the statistical analysis, we kept only the last visit of each patient. Group differences were examined using Wilcoxon rank sum test, also known as Mann Whitney U test, which is a popular nonparametric test to compare outcomes between two independent groups. The effect size (r = z/√N) was calculated with the rstatix package^88^. A Chi-square test examined the difference between two categorical variables. We used standardized Pearson residuals to show the strength and direction of the association between topic and sex. To show topic and PMH association, we first performed a chi-square test of each keyword with all the topics and kept only cases with a p-value < 0.05 and standardized Pearson residual >  = 2. A residual greater than |2| indicates an association, and a residual greater than |4| represents a high association. We used the R software version 4.1.2^89^ for all the statistical analyses. The statistical significance (p) of all the tests was determined at p 0.05. Effect sizes of r 0.1– < 0.3 were considered a small effect, 0.3– < 0.5—a moderate effect, and >  = 0.5 a large effect.

### Schematic description of methods

**Figure 2:**
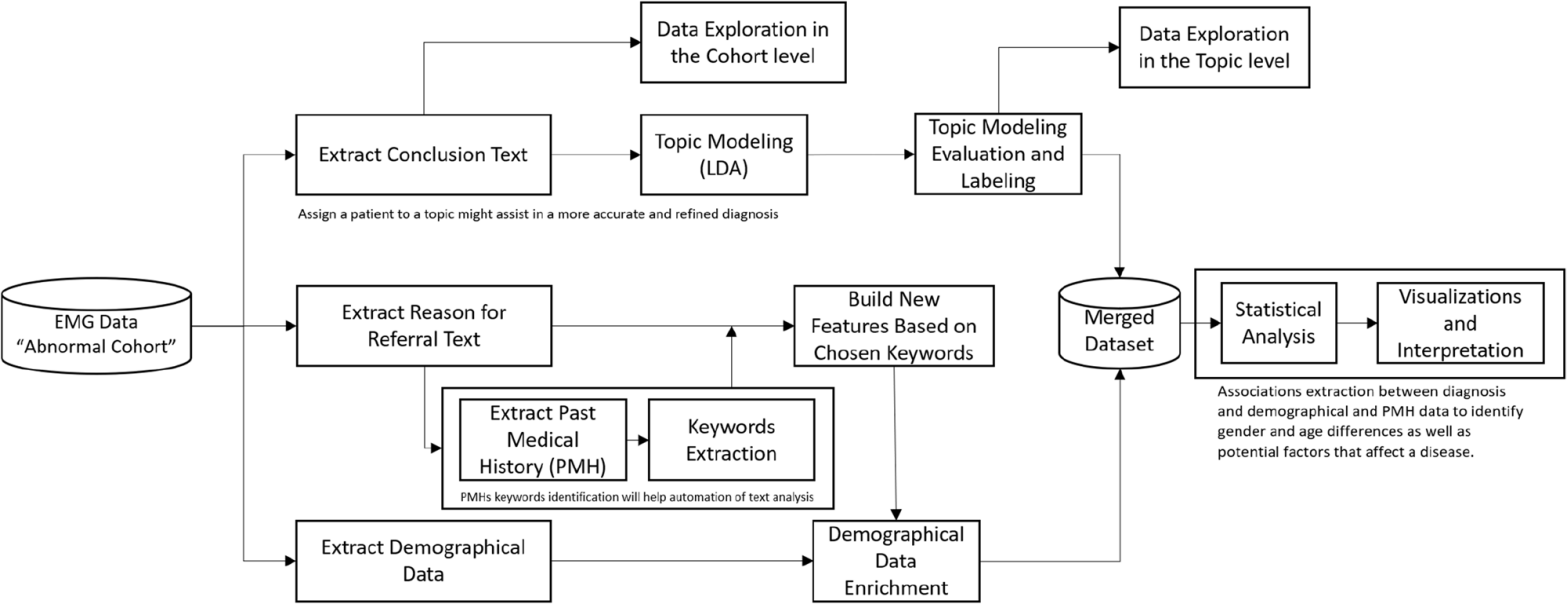
Research methodology schema.

In this work, we integrated several methods to acquire a unique incorporated analysis of EDX clinical notes. Figure 2 displays all the stages of the research methodology schema.

Initially, we created an “abnormal cohort” by filtering out all the study reports with normal results. Next, we extracted the reason for referral and the visit's conclusions from the examination report. The reason for referral provided the patient's past medical history and a keyword extraction algorithm was applied to this sub-cohort (reports containing "history of…"). After creating a keyword list, we put together the new features (PMH indicators) on the “abnormal cohort”. In parallel, we grouped the patients according to the topic modeling of the conclusions text. Following the topic evaluation and initial interpretation, we merged the two datasets. The PMHs and demographical data together with the topics allowed us to perform a statistical analysis that yielded new insights and hypotheses. Additional visualizations summarized and displayed the results in a simple and clear way (Figs. 10, 11).

### Ethical approval

This study was reviewed and approved by the Sheba medical center (SMC) institution review board (9127-22-SMC). We confirm that all experiments were performed in accordance with relevant guidelines and regulations. The data was de-identified and therefore the IRB-Helsinki committee (Sheba medical center international review board for human and animal trials) waived the need for informed consent.

## Results

### Cohort demographical characteristics

**Figure 3:**
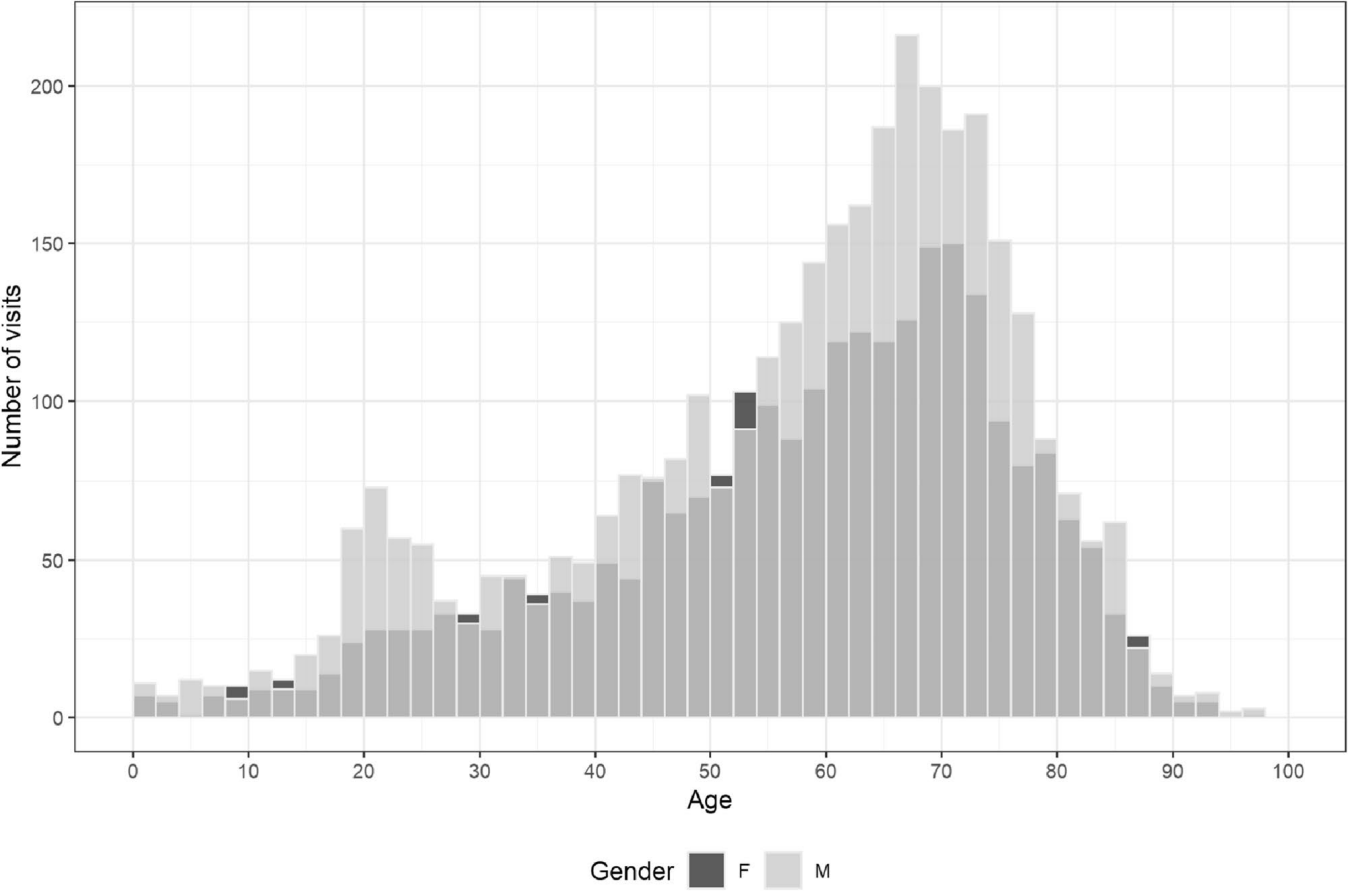
Age-sex distribution of the study cohort (N = 6096).

In the present study, we identified 5601 patients who showed abnormal results in 6575 visits. After excluding visits for which there was no textual summary or conclusions, we were left with 6096 visits, of which 3512 (58%) were male patients and 2584 (42%) were female. Table 1 summarizes the basic demographics of our cohort. In our visit cohort, the number of males was more significant statistically than that of females compared to the entire referral cohort (p < 0.001). Figure 3 displays age and sex histograms. Our target population mainly comprised elderly people aged 60 to 75 with some increase in younger ages (18–26).

**Table 1:** Baseline demographics of our cohort.

Variable	Male	Female	Total	P-value
Number of visits	3512 (58%)	2584 (42%)	6096	P < 0.001
Number of patients	3049 (58%)	2225 (42%)	5274	P < 0.001
Mean age	57.27 (± 19)	57.9 (± 18.1)	57.5 (± 18.6)	0.65

### Textual analysis of the cohort

**Figure 4:**
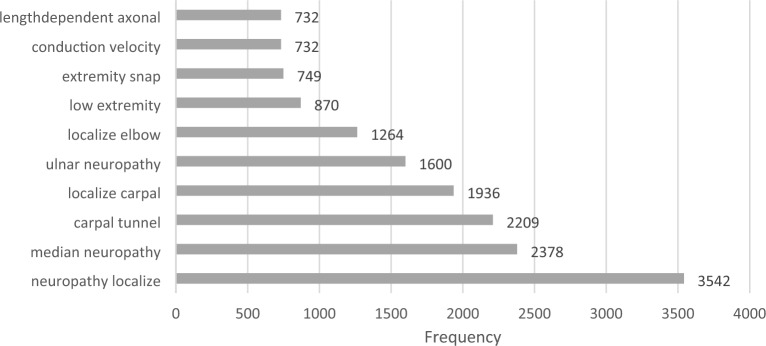
Top-10 Bigrams in the cohort.

A primary text analysis performed on the textual visit summaries aimed to identify frequent words. Figure 4 displays the most frequent bigrams, indicating that the most frequent neuropathy in the corpus is the carpal tunnel syndrome, and the second most frequent is ulnar neuropathy localized at the elbow. This alignes with the literature^90^.

**Figure 5:**
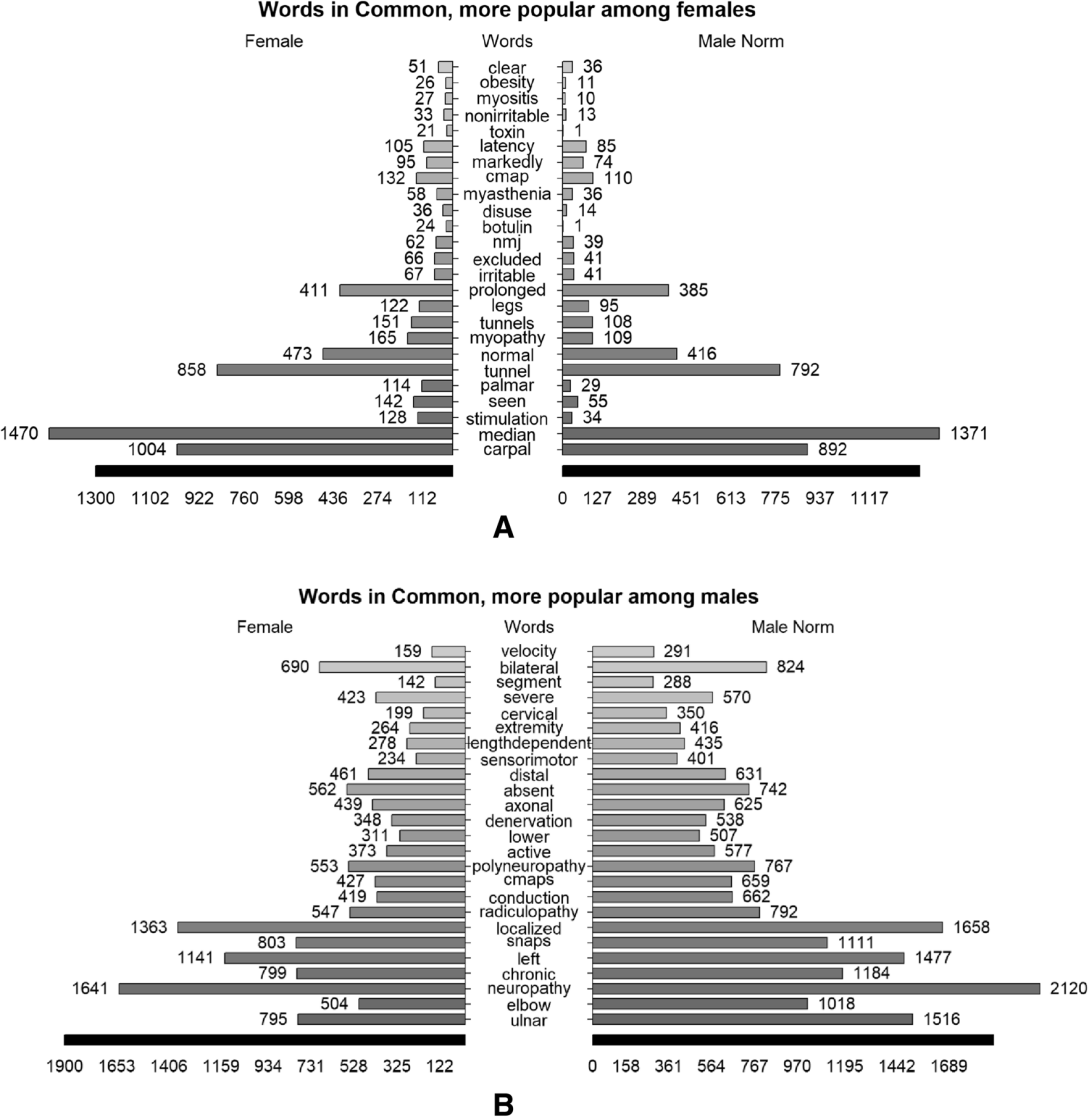
(**A**) Pyramid plot of top 25 words with higher proportion among females. The value on the bar shows the frequency and the normalized frequency for males. (**B**) Pyramid plot of top 25 words with higher proportion among males. The value on the bar shows the frequency and the normalized frequency for males.

Next, we grouped the corpus by sex and analyzed the occurrence rates of identical words among males and females (Fig. 5). This revealed that males and females suffer both from median carpal tunnel syndrome, but it is more common in females (Fig. 5A). Ulnar, elbow, neuropathy, axonal, bilateral, chronic, length-dependent, and cervical were more frequent among males. The words NMJ, myasthenia, botulin, toxin, and obesity were more frequent among females. Radiculopathy appeared in both sexes but was more common in males. In addition, a chi-square test was performed to highlight the statistically significant differences. The results appear in Appendix C.

### Topic modeling result

As detailed in the “Methods” section, we explored several topic modeling algorithms: BERTopic, Top2Vec and Gibbs LDA. The preprocessing stage was largely similar, excluding stopwords removal and lemmatization that were omitted in BERTopic and Top2Vec as per their documentation. BERTopic and Top2Vec with pre-trained embeddings yielded one large topic and many small topics, exceeding 70 in count. Notably, the largest topic in BERTopic was labeled -1, denoting that multiple documents were assigned to the noise topic. Top2Vec using doc2vec embeddings generated topics that were more balanced in size albeit still numerous. Both tools automatically determined the number of topics, often yielding a multitude of small topics. To address this, both algorithms offer a method to reduce and merge similar topics, provided the desired number of topics is specified. However, one limitation of Top2Vec with doc2vec embeddings is its non-deterministic behavior; each run produces different outcomes, and currently, there is no option to ensure consistent results across runs. Appendix H showcases the Top2Vec outcomes using doc2vec embeddings. Furthermore, we explored Gibbs LDA across three configurations, unigrams, bigrams and their combined form. Following a manual evaluation of the outcomes, we opted to proceed with the bigrams configuration. To evaluate the number of topics, our approach began with cross-validation over a broad range of topics, calculating perplexity on validation sets. The cross-validation results are displayed in Appendix A, where stability is evident within the 25–40 range. To choose a definitive outcome, we computed additional evaluation metrics (see Appendix B). The log-likelihood plot (see Appendix B) guided us to two plausible divisions: 25 and 28. Ultimately, a qualitative assessment coupled with consultation from a neurologist led us to select the 25-topic division. We avoided a finer subdivision due to the intricate nature of the analysis and the aim to prevent working with overly small groups during subsequent statistical testing.

**Figure 6:**
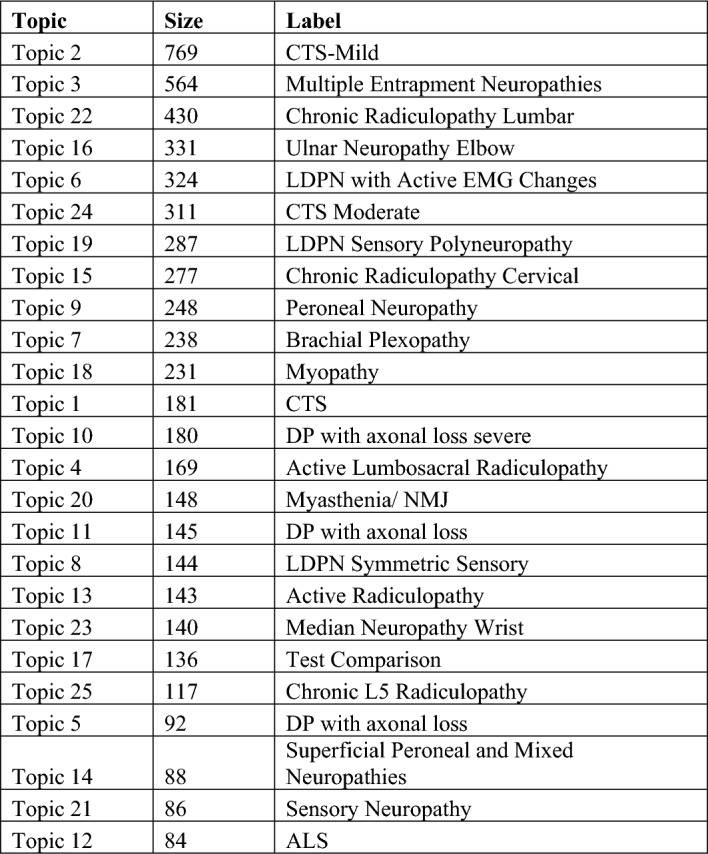
The 25 topics, their incidence and label. A more detailed table featuring the ten most representative bigrams appears in Appendix D.

Figure 6 shows the 25 topics that were yielded. It denotes the topic size and the topic label. The topics ten most characteristic words appear in Appendix D. A neurologist labeled each of the topics based on the words that characterize each topic. The labeling aims to describe the central theme of the respective topic. However, it’s important to note that there are instances where topics may not be entirely coherent, and there might be phrases that do not align perfectly with the assigned label. Topic 2—CTS-Mild, the most recurrent one, features 769 visits; topic 12—ALS is the least frequent with 84 visits only. It is worth noting that there are a few cases where a topic contains several subgroups of patients with somewhat different diagnoses and cases where a topic contains patients that have multiple diagnoses. A neurologist can identify these cases by observing the top terms that describe the topic and suggest if further action is required. Topic 17 (Test comparison) regards, as its name indicates, patients who underwent several tests.

### Age and sex and clinical differentiation

The topics' analysis followed patients’ demographic data and PMH, thus enhancing topic interpretation, knowledge discovery, and hypothesis generation.

**Figure 7:**
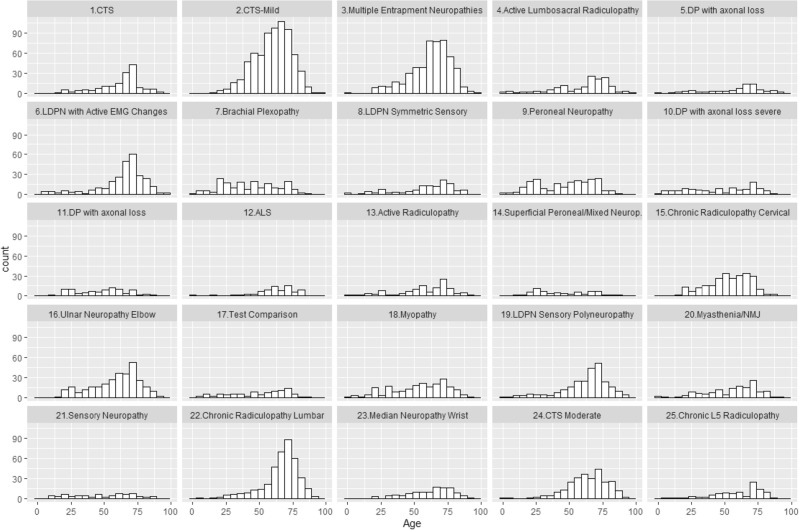
Age distribution by topic.

*Age distribution:* Initially, we analyzed topics by age groups to find the age distribution of each topic (Fig. 7). Several topics were relevant across all ages (e.g., 11—DP with axonal loss). Others were common in younger age groups (e.g., 7—brachial plexopathy, 14—superficial peroneal/ mixed neuropathies). Yet others were more prominent in older ages (e.g., 6—LDPN with active EMG changes, 22—chronic radiculopathy lumbar) or middle age (e.g., 24—CTS moderate, 15—chronic radiculopathy cervical). Notably, some topics peaked at different ages (e.g., 9—peroneal neuropathy, 14—superficial peroneal/ mixed neuropathies).

**Figure 8:**
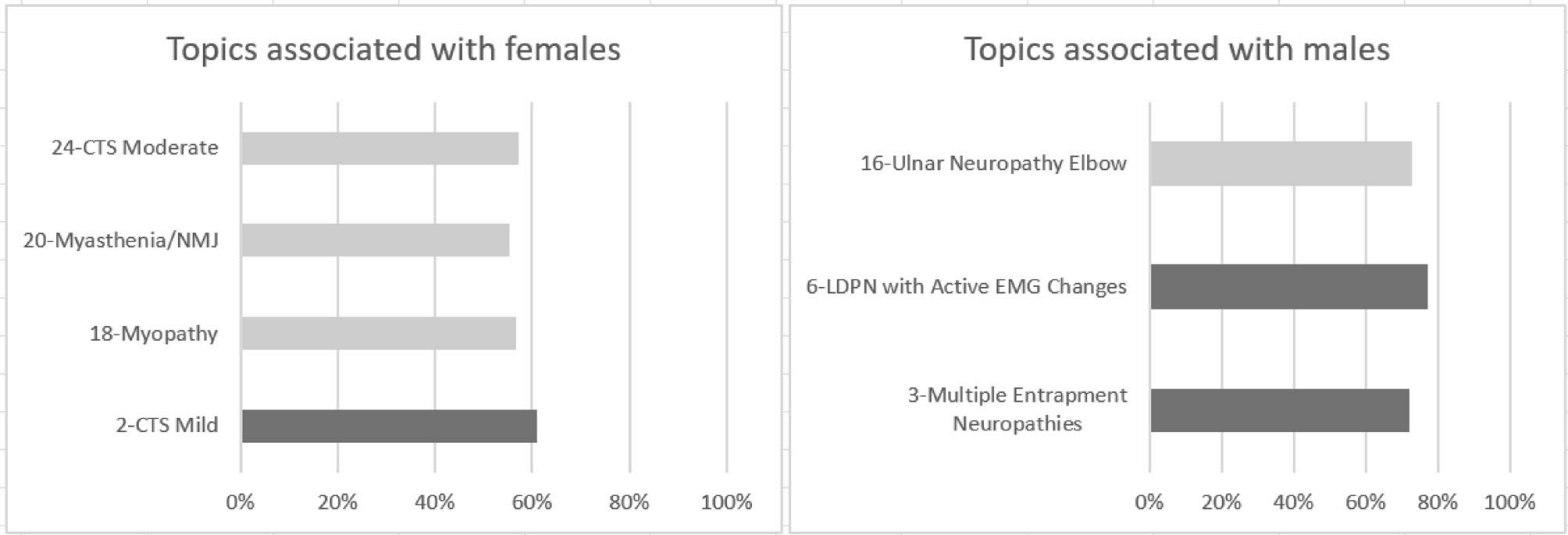
Topic and sex association. In light gray, standardized Pearson residuals greater than 2; in dark gray, standardized Pearson residuals greater than 4, indicating a strong association. The X-axis represents the percentage of females/ males within the topic.

*Sex differences:* Fig. 8 shows a strong association of topic 2 (CTS mild) with females and topics 3 (Multiple Entrapment Neuropathies) and 6 (LDPN with active EMG changes) with males. The lighter colors show a weaker association. Topics 18 (Myopathy), 20 (myasthenia gravis and NMJ disorders), and 24 (CTS moderate) are associated with females, while topic 16 (ulnar neuropathy elbow) —is associated with males.

The simultaneous testing of age and sex distributions also showed that several topics are more frequent at certain ages in females and at other ages in males. Table 2 shows statistically significant age and sex differences (p < 0.05) in only three of the topics: topic 7 (brachial plexopathy), topic 20 (myasthenia gravis and NMJ disorders), and topic 6 (length dependent symmetric axonal polyneuropathy with active EMG changes).

**Table 2:** Wilcoxon test result to find differences in age between males and females per topic.

Topic	P-value	Effect size	Mean age (SD)	
			Males	Females
7—Brachial Plexopathy (N = 187, M = 106, F = 81)	0.0047**	0.206	39.4 (± 19.5)	48 (± 21)
20—Myasthenia/NMJ (N = 139, M = 62, F = 77)	0.0199*	0.197	60.1 (± 18.8)	53.5 (± 18.7)
6—Length Dependent Symmetric Axonal Polyneuropathy with Active EMG Changes (N = 273, M = 211, F = 62)	0.0492*	0.119	65.2 (± 16.6)	59.7 (± 20.1)

*Only statistically significant results are shown. *Last visit per patient was used.

**Figure 9:**
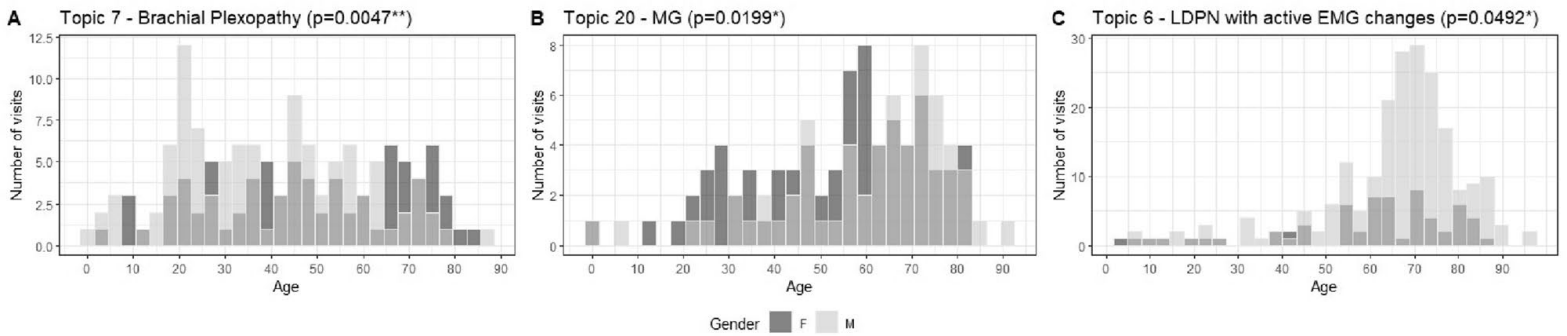
Age distribution by sex of the three topics with statistically significant differences.

Figure 9 shows the age distribution by sex. We found brachial plexopathy more frequent in young males (18–25) and common in females aged 70 years. Myasthenia gravis and NMJ Disorders were more common in females aged 55 to 60 and older males (aged 75). In topic 6 (LDPN with active EMG changes) the majority are males with the highest incidence aged around 70.

### Topics and past medical history

**Figure 10:**
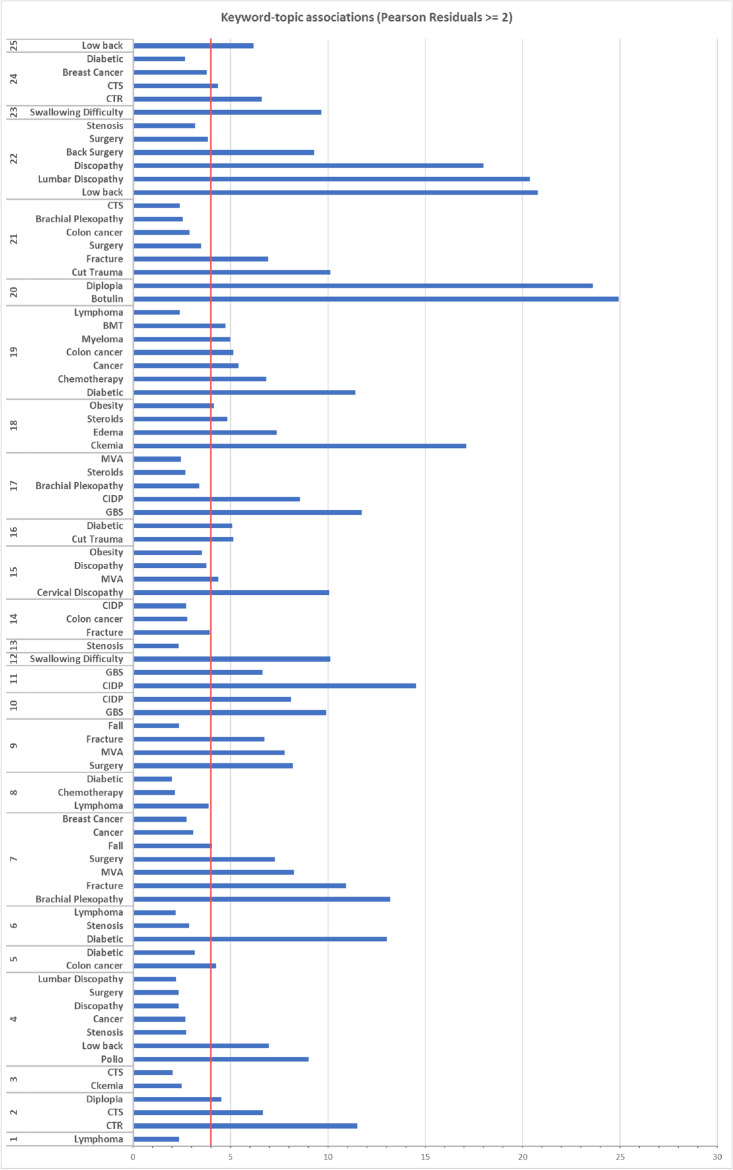
Keyword-topic associations. A keyword appears in the chart under a specific topic if the Pearson residuals are greater than or equal to 2 and the chi-square test is significant. The red target line indicates high association (R = 4).

After the keyword extraction stage, we were left with 37 keywords (see Appendix E and F), which we added to the data as new binary attributes. A Pearson Chi Square Residuals Analysis examined the association between each topic and binary attribute. Of the resulting 925 association tests (37 × 25), 83 emerged as significant (p < 0.05) with a residual >  = 2. After removing the non-significant keywords, 32 keywords were left. The five discarded keywords were fixation surgery, lung cancer, multiple sclerosis, Parkinson, and stroke. To show the main results in a focused, clear and simple way, we generated a keyword-topic association plot that shows topic and PMH associations (see Fig. 10). The results revealed that each topic had at least one associated keyword. While this visualization shows associations the physician is familiar with, it also uncovers previously unknown associations that require further investigation and tracking.

Following are the most prominent associations: topic 20 (Myasthenia Gravis and NMJ Disorders) with botulin and diplopia; topic 22 (Chronic Radiculopathy lumbar) with low back, lumbar discopathy, and back surgery; topic 7 (Brachial Plexopathy) with brachial plexopathy, fracture, MVA, and surgery; topic 6 (LDPN) with diabetes; topic 10 (DP with axonal loss) with GBS and CIDP; Topic 2 (CTS—mild) with CTR and CTS; topic 4 (Active Lumbosacral Radiculopathy) with Polio and low back; topic 18 (Myopathy) with Ckemia and edema; topic 19 (LDPN Sensory Polyneuropathy) with diabetes and chemotherapy; topic 21 (Sensory Neuropathy) with cut trauma, fracture; and topic 12 (ALS) with swallowing difficulty.

**Figure 11:**
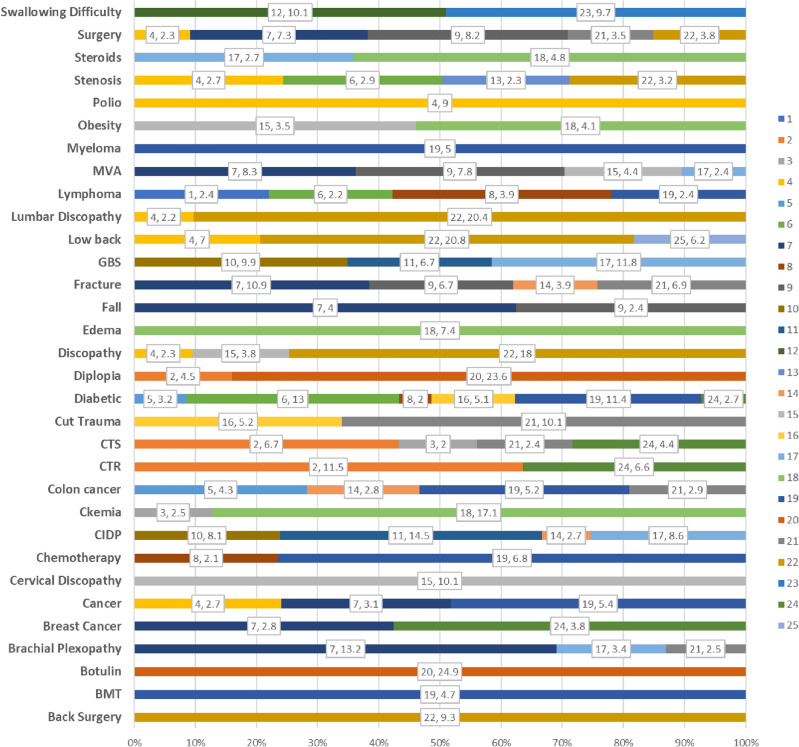
Topics associated with each keyword (proportional view). The chart shows the association only if the Pearson residuals are greater than or equal to 2 and the chi-square test is significant. The label shows the topic number and association level (R).

Figure 11, the keyword-topic association plot, offers another view of the same data, showing the topics each keyword is associated with. We were left with 32 keywords, several keywords are associated with one topic only (e.g., back surgery, BMT, botulin), and others with several topics (e.g., brachial plexopathy, breast cancer). The length of each topic color on the bar represents the strength of the association relative to the other topics on the same bar. For example, the keyword MVA (motor vehicle accident) associates with four topics—topic 7 (brachial plexopathy, R = 8.3), topic 9 (peroneal neuropathy, R = 7.8), topic 15 (chronic radiculopathy cervical, R = 4.4), and topic 17 (test comparison, R = 2.4).

**Figure 12:**
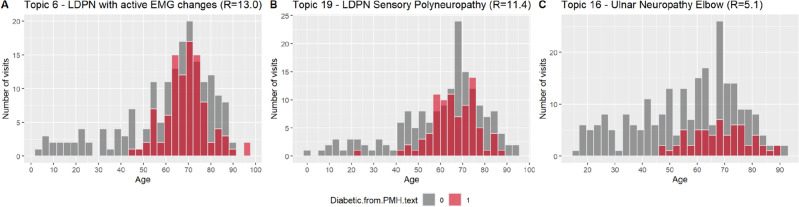
Age distribution by the appearance of diabetes in the PMH text in the three topics with the highest association with diabetes. R indicates the Pearson residual value.

In our cohort, 10% of the patients had a history of diabetes described in the examination report. It was the most frequent PMH item and therefore we examined those patients further against the different topics. Figure 12 shows the histograms of the three topics that had the highest association with diabetes. All of them show that most of the diabetic patients (in red) were 40 or older, the majority being between 60 and 78 years of age. Topic 6 (LDPN with active changes), which has the highest association with DM, comprises mostly males (76.5%) many of whom are diabetic^91^.

## Discussion

In this study, we evaluate the utility of text mining (TM) in clinical practice, specifically in the context of electronic medical record reports of EDX examinations. Our results demonstrate that implementing TM can facilitate a support framework for patients and healthcare providers in the settings of a neuromuscular lab, leading to improved quality of care, diagnosis accuracy, and disease prevention efforts.

We propose a systematic methodology for analyzing EDX evaluation reports using a combination of TM techniques, statistical methods, and easy to capture visualizations. Keyword extraction enriched the data with relevant past medical history. To analyze the corpus as a whole and examine prominent sex differences we used frequency analysis and statistical tests. Topic modeling served to group together patients with similar diagnoses. Pearson Chi-Square Residuals Analysis found associations between topics and PMH, and a Wilcoxon test examined age and gender differences within topics.

Our approach has several advantages (Fig. 1). On the visit level, evaluating the patients enables assigning them automatically to patient groups with similar characteristics. The system would then call the physician's attention to similar cases, associated PMHs, frequent diagnoses, and treatments of other patients in this group. This information may also guide the physician while interviewing the patient and assist in formulating a differential diagnosis. On the research level, our approach promises to generate new hypotheses that would guide future experiment designs.

In our study, 25 topics emerged, and each was labeled based on the ten top bigrams with the highest probability of association with this topic. The topics were analyzed against demographic data such as age and sex for a finer interpretation and additional insights. In several topics, sex emerged as a decisive factor. Four topics were associated with females and three with males. These associations matched other large-scale prevalence studies. For example, one of the female-related topics was carpal tunnel median neuropathy (carpal tunnel syndrome, CTS) which matched general population studies showing higher prevalence in females^92^. Another one was myasthenia gravis (MG) and NMJ disorders (topic 20). MG is an autoimmune disease common in young females. Myopathy (Topic 18), a general term for a pathology in the skeletal muscles, displayed similar results; Two of the four main myopathies, polymyositis and dermatomyositis are more common in females^93^.

We also found clinical conditions that were more common in males, such as length-dependent polyneuropathy (LDPN) with axonal damage and denervation (topic 6) (Fig. 8). Several studies have found that diabetic sensorimotor polyneuropathy (DSP) is more prevalent in males^94,95^.

Reportedly, up to 7% of the overall neuropathy prevalence occurs in older patients^96^. Different conditions relate to different ages. For example, lumbar radiculopathy symptoms typically begin in midlife, with men often affected in their 40 s while women in their 50 s and 60 s^97,98^. Elderly people are the largest group with lumbar spine diseases^99^. Carpal tunnel syndrome (CTS) is the most frequent compression neuropathy, usually occurring after the age of 30; 76% of the patients become symptomatic between the age of 40–70^100^. In contrast, chronic inflammatory demyelinating polyneuropathy (CIDP) can occur throughout life from childhood to old age^101^.

Our age distribution analysis (Fig. 7) showed that topic distribution differs in different ages. For example, chronic lumbar radiculopathy (topic 22) is more common in older persons, while carpal tunnel syndrome (topics 1, 2, 24) spans a wider age range. On the other hand, brachial plexopathy (topic 7) is also common among young adults (in their 20 s). DP with axonal loss (topic 11) is common among this age group as well as in elderly people. This is also supported in the literature^101,102^. Observing the age distribution of a specific topic can confirm previously known phenomena or reveal new hypotheses.

Age differences combined with the sex layer are particularly interesting as certain phenomena occur in females before they occur in males and vice versa. In this study, we found three topics (Table 2) with statistically significant differences between male and female ages. The most significant difference (p < 0.01) was found in Brachial Plexopathy (topic 7) where males were younger than females^103^. Myasthenia Gravis and NMJ Disorders (topic 20) also revealed differences (p < 0.05), showing that females suffer from myasthenia gravies at an earlier age than males. This agrees with the findings of Grob et al.^44^ who showed that myasthenia gravis is more common in younger females and older males.

A patient’s past medical history is a powerful diagnostic tool. It should be given special consideration when seeking an appropriate balance between medical history and other diagnostics. Creating statistically adjusted combinations of information based on medical histories in daily practice is challenging. We have hypothesized that developing a rational approach to the use of medical history could improve diagnostic efficiency and clinical effectiveness, which would benefit the individual patient and the treating physician. To facilitate a better PMH investigation we performed a data enrichment process and extracted additional features from the examination report texts. Our keywords extraction method yielded keywords and binary features of selected keywords indicated whether a patient had a previous medical condition or not. Examination of the associations between topics and PMHs revealed previously known associations but also less obvious ones that require further investigation.

Strong associations emerged between topics and PMHs (see Fig. 11,12). The most significant (highest R value) was between topic 20 (myasthenia gravis and NMJ Disorders) and botulinum. Botulinum toxin injections are known to cause neuromuscular changes^104^. Patients with diseases of the NMJ are clinically similar to MG patients, making it difficult to exclude MG in those patients based on an EDX examination. Weaker associations (2 <  = R < 4) were also found useful. One example was the association between breast cancer and brachial plexopathy (topic 7), also conforming to the literature^56,105,106^. The second topic associated with breast cancer is CTS, also mentioned in the literature^107^.

Diabetes mellites was the most prevalent PMH in our dataset. Of the 25 topics, only 3 had a strong association with DM. The strongest association was with topic 6 (LDPN) (Figs. 10, 11, 12). Length-dependent polyneuropathy is a known complication of diabetes^48–51^ and this association is not surprising. The majority of patients in this topic were males (76.5%).

An association between Diabetes and CTS (R = 2.6) was found only in one out of the three CTS topics, topic 24 (Figs. 10, 11). This might suggest that other additional factors connect diabetes to carpal tunnel syndrome, which requires further research.

Topic 19, length dependent sensory neuropathy, showed a close association with chemotherapy and cancer, and is known as chemotherapy-induced peripheral neuropathy (CIPN)^108^. On the other hand, Topic 6, which also comprises length-dependent polyneuropathy (LDPN) patients, had no association with chemotherapy, probably because chemotherapy affects sensory nerves more than motor nerves^109,110^. We also found a close association between topic 18, myopathy, and elevated serum creatine kinase (CK). Support for this finding appears in the literature which mentions that CK levels increase in cases of muscle pathology^111,112^.

## Limitations and future research

The study has several limitations. The first is a selection bias since physicians do not always write down the exact past medical history (PMH) for each of the patients or decide ad hoc what deserves documenting. Our dataset leans on the reports of several physicians, thus somewhat attenuating this drawback. Except for the differences in PMH documentation, each physician may use distinct terminology to depict identical phenomena. Demographics, geographical location, and even prior daily activities can affect their lexical preferences^113,114^. In our study, all participating physicians are affiliated with the same department and generally adhere to consistent technical terminology, although certain disparities persist. In situations where there is an abundance of data accessible, there are occurrences where physicians utilize various forms of the same term within a single report. For example, a word and its abbreviation could be enclosed within parentheses. This practice enhances the topic modeling algorithm's capability to detect consistent topics, even when diverse terminologies are used. This limitation diminishes even further when word embeddings are employed for topic modeling. Another limitation is that the study involved a single medical center. While all the textual reports followed the same format, this fact may impede generalizing the findings to other clinical reports, patient cohorts, and institutions. As part of this work's preprocessing stage, we defined several rules to correct often misspelled terms and uniform expressions to identify past medical history and negation (e.g., "there is no evidence"). It will probably be necessary to adjust the preprocessing step for a different dataset and context.

Topic modeling is an unsupervised technique to classify patients. The classification is not deterministic and therefore harder to evaluate. It is recommended to test several techniques and parameters until a stable and desirable model is reached. Furthermore, labeling and interpreting the topics is also not deterministic. If we only consider the top words representing the topic, it's unclear whether these words convey the same theme related to a specific diagnosis, whether they emerge due to a patient having multiple diagnoses, or if the topic encompasses multiple merged sub-topics. To address this limitation, we recommend examining the documents within each topic and verifying the relevance of the words used to formulate the topic label, including checking the frequency of the top words.

This study focuses on the analysis of the EDX report. A future study could include additional information sources and integrate past medical histories documented in the patient's medical records. Furthermore, in a future study, there could be an investigation into the evolution of topics, tracking their changes over time, identifying emerging phenomena, recognizing topics and PMHs that appear to become more frequent and identifying emerging trends. In this study, our focus was on the cohort with diagnosed abnormalities. Future research can encompass the entire population, extract past medical histories, and explore differences in sex and age among each cohort.

## Conclusion

The current study demonstrates that text mining is a useful tool for free-text data analysis, such as medical histories in electronic medical records (EMRs). It assists in making accurate diagnoses and developing hypotheses based on past medical history, taking into account age- and sex-related conditions. Despite certain limitations, the proposed method and findings can potentially enhance healthcare information systems in research scenarios and at the point of care.

### Supplementary Information

Supplementary Information.

## Data Availability

Upon a reasonable request and with the approval of the Sheba Hospital review board we will consider sharing the data. To submit a request please contact Dr. Shelly (SS).

## Code availability

The code used for conducting the topic modeling and generating the results presented in this paper is openly available at the following URL: https://github.com/ronirg/EMG-TM-Paper-2023. The dataset found in the repository was manually generated specifically for running the code; it is not a real dataset.
