# Supplementary material for: Towards a practical use of text mining approaches in electrodiagnostic data
Source: Sci Rep. 2023 Nov 9;13:19483. doi: 10.1038/s41598-023-45758-0 (PMC10636146; doi:10.1038/s41598-023-45758-0)
Supplement: Supplementary file 1 — Supplementary Information. [file 41598_2023_45758_MOESM1_ESM.docx]

**Appendices**

**Appendix A:** Cross-validation of topic modeling

**
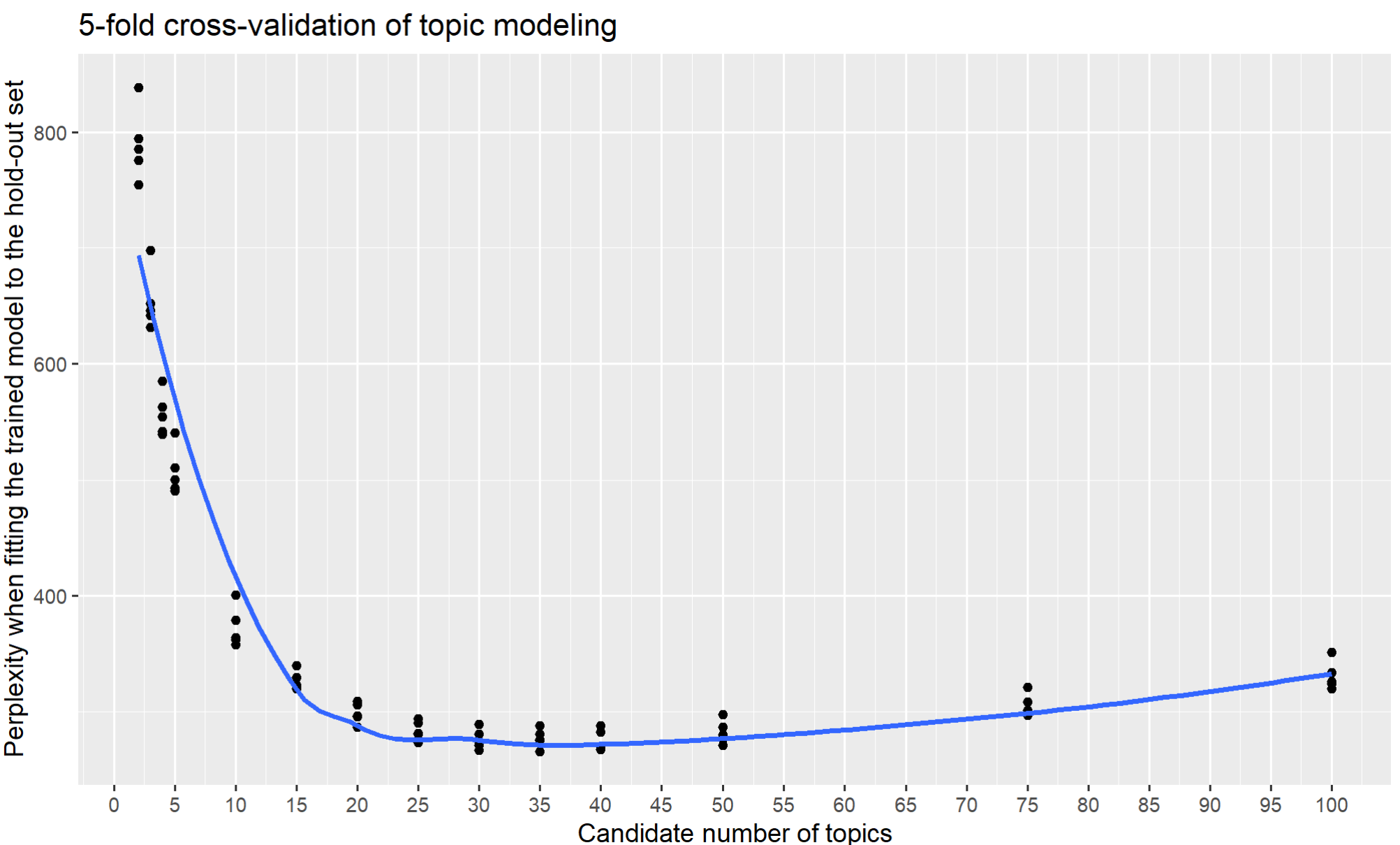
**

**Appendix B:** Ldatuning plot to assess the number of topics


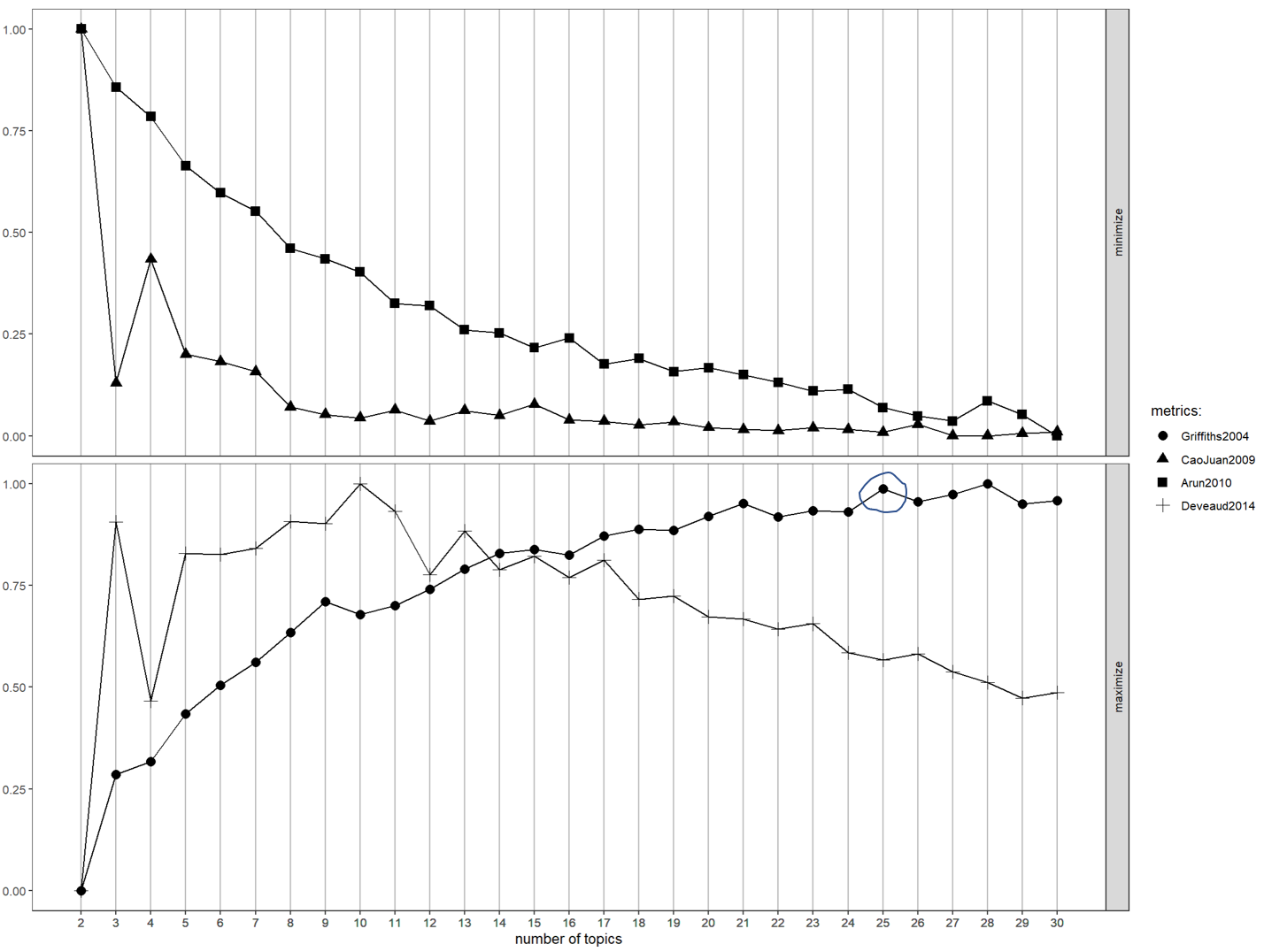


**Appendix C:** Terms with significant sex differences (p<0.05). It doesn’t include terms that appeared in one gender and didn’t appear in the other.

1. Higher proportion among females

|  | **Term** | **Female** | **Male** | **p_value** | **Female Rate** | **Male Rate** |
| --- | --- | --- | --- | --- | --- | --- |
| 1 | median | 1266 | 1628 | 0.013 | 56.9% | 53.4% |
| 2 | carpal | 896 | 1087 | <0.001 | 40.3% | 35.7% |
| 3 | tunnel | 770 | 968 | 0.031 | 34.6% | 31.7% |
| 4 | normal | 413 | 491 | 0.021 | 18.6% | 16.1% |
| 5 | myopathy | 140 | 128 | <0.001 | 6.3% | 4.2% |
| 6 | tunnels | 130 | 128 | 0.008 | 5.8% | 4.2% |
| 7 | seen | 124 | 69 | <0.001 | 5.6% | 2.3% |
| 8 | stimulation | 115 | 44 | <0.001 | 5.2% | 1.4% |
| 9 | legs | 106 | 110 | 0.043 | 4.8% | 3.6% |
| 10 | palmar | 104 | 36 | <0.001 | 4.7% | 1.2% |
| 11 | excluded | 62 | 50 | 0.006 | 2.8% | 1.6% |
| 12 | nmj | 58 | 52 | 0.030 | 2.6% | 1.7% |
| 13 | irritable | 57 | 49 | 0.019 | 2.6% | 1.6% |
| 14 | myasthenia | 56 | 45 | 0.009 | 2.5% | 1.5% |
| 15 | gravis | 39 | 31 | 0.029 | 1.8% | 1.0% |
| 16 | disuse | 29 | 18 | 0.010 | 1.3% | 0.6% |
| 17 | nonirritable | 28 | 16 | 0.006 | 1.3% | 0.5% |
| 18 | obesity | 25 | 15 | 0.014 | 1.1% | 0.5% |
| 19 | botulin | 24 | 2 | <0.001 | 1.1% | 0.1% |
| 20 | toxin | 21 | 1 | <0.001 | 0.9% | 0.0% |
| 21 | myositis | 21 | 10 | 0.007 | 0.9% | 0.3% |
| 22 | problem | 20 | 7 | 0.002 | 0.9% | 0.2% |
| 23 | ncs | 17 | 8 | 0.016 | 0.8% | 0.3% |
| 24 | usec | 17 | 8 | 0.016 | 0.8% | 0.3% |
| 25 | injection | 15 | 1 | <0.001 | 0.7% | 0.0% |
| 26 | treatment | 11 | 3 | 0.013 | 0.5% | 0.1% |
| 27 | antidromic | 8 | 1 | 0.012 | 0.4% | 0.0% |

1. Higher proportion among males*

|  | **Term** | **Female** | **Male** | **p_value** | **Female Rate** | **Male Rate** |
| --- | --- | --- | --- | --- | --- | --- |
| 1 | neuropathy | 1444 | 2536 | <0.001 | 64.9% | 83.2% |
| 2 | localized | 1198 | 2007 | <0.001 | 53.8% | 65.8% |
| 3 | ulnar | 664 | 1802 | <0.001 | 29.8% | 59.1% |
| 4 | left | 990 | 1712 | <0.001 | 44.5% | 56.1% |
| 5 | chronic | 704 | 1409 | <0.001 | 31.6% | 46.2% |
| 6 | snaps | 673 | 1313 | <0.001 | 30.2% | 43.1% |
| 7 | right | 850 | 1283 | 0.005 | 38.2% | 42.1% |
| 8 | elbow | 436 | 1238 | <0.001 | 19.6% | 40.6% |
| 9 | bilateral | 613 | 990 | <0.001 | 27.6% | 32.5% |
| 10 | radiculopathy | 491 | 942 | <0.001 | 22.1% | 30.9% |
| 11 | absent | 475 | 872 | <0.001 | 21.3% | 28.6% |
| 12 | polyneuropathy | 414 | 855 | <0.001 | 18.6% | 28.0% |
| 13 | reduced | 423 | 766 | <0.001 | 19.0% | 25.1% |
| 14 | cmaps | 338 | 764 | <0.001 | 15.2% | 25.1% |
| 15 | conduction | 333 | 752 | <0.001 | 15.0% | 24.7% |
| 16 | distal | 377 | 745 | <0.001 | 16.9% | 24.4% |
| 17 | axonal | 336 | 720 | <0.001 | 15.1% | 23.6% |
| 18 | active | 305 | 669 | <0.001 | 13.7% | 21.9% |
| 19 | severe | 354 | 633 | <0.001 | 15.9% | 20.8% |
| 20 | lower | 249 | 588 | <0.001 | 11.2% | 19.3% |
| 21 | denervation | 273 | 586 | <0.001 | 12.3% | 19.2% |
| 22 | changes | 327 | 523 | 0.018 | 14.7% | 17.2% |
| 23 | lengthdependent | 213 | 508 | <0.001 | 9.6% | 16.7% |
| 24 | extremity | 215 | 487 | <0.001 | 9.7% | 16.0% |
| 25 | moderate | 287 | 472 | 0.009 | 12.9% | 15.5% |
| 26 | sensorimotor | 173 | 452 | <0.001 | 7.8% | 14.8% |
| 27 | abnormalities | 202 | 441 | <0.001 | 9.1% | 14.5% |
| 28 | cervical | 183 | 414 | <0.001 | 8.2% | 13.6% |
| 29 | muscles | 245 | 406 | 0.013 | 11.0% | 13.3% |
| 30 | lumbar | 239 | 399 | 0.011 | 10.7% | 13.1% |

*The table shows the top 30 terms.

**Appendix D:** The 25 topics with the top 10 bigrams that describe each topic.

| **Topic 1 (181)** | **Topic 2 (769)** | **Topic 3 (564)** | **Topic 4 (169)** | **Topic 5 (92)** |
| --- | --- | --- | --- | --- |
| **CTS** | **CTS-Mild** | **Multiple Entrapment Neuropathies** | **Active Lumbosacral Radiculopathy** | **DP with axonal loss** |
| tunnel syndrome | carpal tunnel | neuropathy localize | neurogenic change | conduction velocity |
| carpal tunnel | median neuropathy | ulnar neuropathy | normal snap | demyelinating neuropathy |
| neuropathy wrist | localize carpal | localize elbow | distal muscle | severe enough |
| median neuropathy | neuropathy localize | median neuropathy | background normal | mild change |
| ulnar neuropathy | mild median | carpal tunnel | lumbosacral polyradiculopathy | distal latency |
| mild left | right mild | localize carpal | chronic neurogenic | enough suggest |
| syndrome prolong | bilateral mild | left ulnar | ongoing denervation | may due |
| left ulnar | bilateral median | mild ulnar | chronic reinnervation | axon loss |
| mild right | mild left | left mild | muscle right | suggest primary |
| neuropathy due | left mild | mild median | proximal distal | secondary axon |
|  |  |  |  |  |
| **Topic 6 (324)** | **Topic 7 (238)** | **Topic 8 (144)** | **Topic 9 (248)** | **Topic 10 (180)** |
| **LDPN with Active EMG Changes** | **Brachial Plexopathy**** | **LDPN Symmetric Sensory** | **Peroneal Neuropathy** | **DP with axonal loss severe** |
| chronic reinnervation | brachial plexopathy | length dependent | peroneal neuropathy | conduction block |
| distal low | radial neuropathy | sensory motor | neuropathy localize | sensorimotor polyneuropathy |
| dependent pattern | neurogenic change | polyneuropathy small | distal peroneal | demyelinating sensorimotor |
| extremity snap | renervation sign | axonal polyneuropathy | localize fibula | can compatible |
| length dependent | complite denervation | low extremity | neurogenic change | lengthdependent demyelinating |
| low extremity | spiral groove | motor axonal | conduction block | nonlengthdependent demyelinating |
| cmaps emg | severe left | small absent | neuropathy probably | slow conduction |
| lengthdependent axonal | severe right | dependent polyneuropathy | common peroneal | motor polyneuropathy |
| reinnervation length | complete denervation | conduction velocity | deep peroneal | compatible gbs |
| absent distal | facial neuropathy | axonal length | change ta | prolong latency |
|  |  |  |  |  |
| **Topic 11 (145)** | **Topic 12 (84)** | **Topic 13 (143)** | **Topic 14 (88)** | **Topic 15 (277)** |
| **DP with axonal loss** | **ALS** | **Active Radiculopathy** | **Superficial Peroneal and** **Mixed Neuropathies** | **Chronic Radiculopathy Cervical** |
| conduction block | motor neuron | neurogenic change | superficial peroneal | cervical radiculopathy |
| conduction velocity | motor neuropathy | active denervation | sural snap | chronic cervical |
| compare previous | neuron disease | chronic neurogenic | left peroneal | chronic nonactive |
| demyelinating polyneuropathy | normal snap | mild chronic | peroneal snap | nonactive cervical |
| axonal loss | ongoing denervation | innervate muscle | absent left | right chronic |
| slow conduction | differential diagnosis | active component | tibialis anterior | radiculopathy c7 |
| distal latency | active ongoing | change left | neuropathy absent | left chronic |
| sensory motor | diagnosis include | l5 radiculopathy | peroneal cmaps | radiculopathy c67 |
| small cmaps | body region | low extremity | autonomic neuropathy | meralgia paresthetica |
| primarily demyelinating | identify background | chronic left | peroneal nerve | radiculopathy c5 |
|  |  |  |  |  |
| **Topic 16 (331)** | **Topic 17 (136)** | **Topic 18 (231)** | **Topic 19 (287)** | **Topic 20 (148)** |
| **Ulnar Neuropathy Elbow** | **Test Comparison** | **Myopathy** | **LDPN Sensory Polyneuropathy** | **Myasthenia/NMJ** |
| ulnar neuropathy | comparison previous | irritable myopathy | lengthdependent axonal | disease nmj |
| neuropathy localize | change comparison | can due | extremity snap | myasthenia gravis |
| elbow segment | significant change | due severe | low extremity | neuromuscular junction |
| localize elbow | without significant | spontaneous activity | distal low | nmj myasthenia |
| conduction velocity | improvement comparison | leg snap | absent distal | junction disorder |
| ulnar conduction | worsen comparison | probably due | symmetric lengthdependent | probably due |
| slow ulnar | markedly improvement | leg edema | polyneuropathy absent | clear disease |
| across elbow | polyneuropathy without | absent leg | sensorimotor polyneuropathy | significant decrement |
| velocity across | residual change | needle exam | axonal sensorimotor | abnormal jitter |
| reduce ulnar | without change | clinical correlation | sensory polyneuropathy | nmj disorder |
|  |  |  |  |  |
| **Topic 21 (86)** | **Topic 22 (430)** | **Topic 23 (140)** | **Topic 24 (311)** | **Topic 25 (117)** |
| **Sensory Neuropathy** | **Chronic Radiculopathy Lumbar** | **Median Neuropathy Wrist** | **CTS Moderate** | **Chronic L5 Radiculopathy** |
| ulnar neuropathy | lumbar radiculopathy | localize wrist | carpal tunnel | chronic reinnervation |
| motor involvement | radiculopathy l5 | median neuropathy | support come | normal snap |
| without motor | chronic nonactive | neuropathy localize | localize carpal | muscle share |
| median neuropathy | chronic active | support come | neuropathy localize | background normal |
| sensory ulnar | left chronic | come prolong | median neuropathy | reinnervation muscle |
| neuropathy without | radiculopathy s1 | wrist support | median snap | share myotome |
| mild sensory | nonactive lumbar | sensory median | snap prolong | radiculopathy chronic |
| radial neuropathy | chronic lumbar | median latency | tunnel support | myotome background |
| neuropathy probably | mild chronic | right median | reduce median | ongoing denervation |
| left sensory | active lumbar | left median | motor latency | active ongoing |

*The lemma of left is leave and was changed to left to avoid confusion in the interpretation. The term “leave” didn’t appear in the dataset.

**Topic 7 mainly included three groups of patients, those with brachial plexopathy, patients with radial neuropathy and a small subset of patients with facial neuropathy.

**Appendix E:** TFIDF keywords. V indicates that a term was included in the association analysis. Three keywords (“swallowing difficulty”, “diplopia”, and “edema”) were not identified automatically due to low frequency and were added manually to the list.

| **Term** | **TFIDF weight** | **Included in analysis** |
| --- | --- | --- |
| dm | 746.5843122 | ✓ |
| surgery | 331.5749723 | ✓ |
| ctr | 276.1409111 | ✓ |
| mva | 245.6820981 | ✓ |
| lumbar | 240.1135417 | lumbar discopathy |
| trauma | 221.3558656 |  |
| discopathy | 220.0078405 | ✓ |
| ca | 214.6869615 | ✓ |
| fracture | 206.5321371 | ✓ |
| cervical | 202.3906438 | cervical discopathy |
| polyneuropathy | 185.7267789 |  |
| chemotherapy | 182.5245415 | ✓ |
| leg | 174.0397072 |  |
| gbs | 157.2085102 | ✓ |
| severe | 155.523968 |  |
| bilateral | 146.2203452 |  |
| cts | 143.9553509 | ✓ |
| back | 137.0571079 | low back |
| low | 134.2381769 |  |
| pain | 131.3908869 |  |
| fall | 129.2776272 | ✓ |
| arm | 127.3233756 |  |
| treatment | 123.0683963 |  |
| pd | 119.1713245 | ✓ |
| mm | 109.4703032 | ✓ |
| shoulder | 104.1106308 |  |
| stroke | 101.5413392 | ✓ |
| cidp | 101.5329324 | ✓ |
| hand | 100.6097023 |  |
| breast | 100.05101 | ✓ |
| progressive | 96.56740962 |  |
| foot | 92.17980055 |  |
| lymphoma | 91.30990717 | ✓ |
| neuropathy | 89.87460259 |  |
| ms | 87.36815391 | ✓ |
| delivery | 86.73708111 |  |
| spinal | 84.95164685 |  |
| wrist | 82.33522661 |  |
| lung | 80.4307901 | lung cancer |
| drop | 78.80976695 |  |
| bmt | 77.64046581 | ✓ |
| stenosis | 77.57932714 | ✓ |
| week | 77.00829883 |  |
| cva | 75.50680446 |  |
| disease | 73.09001385 |  |
| obesity | 72.02603372 | ✓ |
| ulnar | 69.28976437 |  |
| polio | 68.34399827 | ✓ |
| numbness | 68.28448574 |  |
| ckemia | 68.04234787 | ✓ |
| elbow | 63.2580346 |  |
| walk | 61.71156345 |  |
| mild | 61.13755483 |  |
| sensorimotor | 61.05332818 |  |
| due | 60.16353733 |  |
| fixation | 59.56450154 | fixation surgery |
| forearm | 58.50464575 |  |
| radiculopathy | 56.76861279 |  |
| chf | 56.28610564 |  |
| multiple | 54.67982747 |  |
| recurrent | 54.61266763 |  |
| hemiparesis | 54.24640121 |  |
| dialysis | 53.76591249 |  |
| palsy | 52.74318093 |  |
| axonal | 52.09371407 |  |
| year | 51.3322803 |  |
| ra | 50.68113819 |  |
| cut | 49.05003864 | cut trauma |
| difficulty | 47.73519728 |  |
| limb | 47.61338908 |  |
| loss | 47.3658912 |  |
| ischemic | 47.31245619 |  |
| mg | 46.48106426 |  |
| weight | 46.24407503 |  |
| finger | 46.13373805 |  |
| suspect | 45.47457847 |  |
| injury | 45.32642924 |  |
| damage | 43.83659605 |  |
| sensory | 43.22948062 |  |
| steroid | 42.20279253 | ✓ |
| treat | 42.1322102 |  |
| muscle | 42.0816029 |  |
| syndrome | 39.96556355 |  |
| aml | 39.79411181 |  |
| dislocation | 39.46413999 |  |
| myelitis | 38.95660656 |  |
| amyloidosis | 37.98169029 |  |
| mts* | 37.86128104 |  |
| fmf | 37.56983134 |  |
| humerus | 36.59514433 |  |
| release | 36.337364 |  |
| knee | 36.28331797 |  |
| covid | 36.2651126 |  |
| improvement | 35.7817515 |  |
| heart | 35.55329154 |  |
| small | 34.83212974 |  |
| brain | 34.25360605 |  |
| melanoma | 34.09071634 |  |
| transplantation | 34.06837078 |  |
| hip | 33.96296884 |  |
| colon | 33.80694293 | ✓ |
| ankle | 33.70500478 |  |
| brachial | 33.70314301 | ✓ |
| tetraparesis | 33.35352901 |  |
| fiber | 33.09671304 |  |
| ihd | 32.85663954 |  |
| day | 32.70074765 |  |
| last | 32.28055192 |  |
| pacemaker | 32.08143771 |  |
| bariatric | 31.7198366 |  |
| botulin | 31.57207332 | ✓ |
| plexopathy | 31.4015857 |  |
| multitrauma | 31.1767095 |  |
| nerve | 31.16299362 |  |
| now | 31.01570785 |  |
| proximal | 30.92876128 |  |
| keytruda | 30.44933539 |  |

*The lemmatization function omitted the s in mts therefore we added it and marked in red. We didn’t have ‘mt’ term in the dataset.

**Appendix F:** Frequent bigrams

| **Bigram** | **Frequency** |
| --- | --- |
| low back | 105 |
| lumbar discopathy | 86 |
| fracture surgery | 70 |
| leg s | 66 |
| back pain | 43 |
| drop foot | 42 |
| back surgery | 41 |
| lung ca | 40 |
| spinal stenosis | 40 |
| fixation surgery | 35 |
| axonal polyneuropathy | 34 |
| bilateral cts | 34 |
| ca breast | 34 |
| cut trauma | 32 |
| ulnar neuropathy | 31 |
| ca chemotherapy | 30 |
| cervical discopathy | 30 |
| lumbar surgery | 28 |
| weight loss | 28 |
| bilateral ctr | 26 |

**Appendix G:** Normalized concepts

| **Original format** | **Normalized format** |
| --- | --- |
| bm transplantation | bmt |
| bone marrow transplant | bmt |
| bone marrow transplantation | bmt |
| bottox | botulin |
| botulinum | botulin |
| cancer | ca |
| chemotherapy | chemo |
| diabetes | dm |
| diabetes mellitus | dm |
| gillian barre | gbs |
| guillain barre | gbs |
| guillainbarre | gbs |
| multiple myeloma | mm |
| motor vehicle accident | mva |
| motorbike accident | mva |
| motorcycle accident | mva |
| nsclc | lung ca |
| parkinson | pd |
| parkinsonism | pd |

**Appendix H:** Top2Vec results

The table displays the topics derived from the Top2Vec algorithm, following hierarchical topic reduction resulting in 25 topics. The table is organized by topic size, which indicates the number of documents assigned to each topic. The 'Topic LDA' column corresponds to the topic number associated with the LDA partition mentioned in the paper, demonstrating the highest degree of alignment with the Top2Vec partition. The match rate is computed as the count of documents present in both matched topics, divided by the size of the Top2Vec topic. The topic labels were assigned by considering the matched LDA topic label and reviewing the documents within each topic, while also analyzing the descriptive terms. The Descriptive Terms section presents the top 10 terms generated by Top2Vec to characterize each topic, based on their similarity to the cluster centroid. Any topic label that is new and dissimilar from the labels in the LDA partition in the paper is highlighted in yellow.

While topic 15 aligns significantly with LDA topic 3 (Multiple Entrapment Neuropathies), it's evident that topic 3 was divided into two distinct clusters, namely clusters 15 and 18. Upon reviewing the documents within topic 15, it becomes apparent that the predominant theme revolves around ulnar neuropathy at the elbow. Notably, some of these documents encompass cases of multiple neuropathies. Except for topic 3, the additional topics were divided into two topics in the Top2Vec result: topics 2, 7, 15, 18, and 22, each conveying a more specific interpretation.

| **Topic Top2 Vec** | **Size** | **Topic LDA** | **Match Rate** | **Topic Label** | **Desciptive Terms** |
| --- | --- | --- | --- | --- | --- |
| 0 | 566 | 2 | 0.86 | CTS-Mild | palmar;locazlied;stimulation;carpal;tunnel;localized;lfc;paresthetica;pathologic; |
| 1 | 341 | 22 | 0.92 | Chronic Radiculopathy Lumbar | chornic;radiculopathy;criterion;als;paraspinal;cervical;myotomes;lumbar;stenosis; |
| 2 | 300 | 11 | 0.33 | Sensorimotor Length Dependent Axonal Polyneuropathy | dispersion;velocities;extremities;prolongation;range;mostly;compared;also;acquired; |
| 3 | 297 | 6 | 0.84 | LDPN with Active EMG Changes | well;dependent;length;innervation;re;pattern;symmetric;sensorimotor;as; |
| 4 | 285 | 16 | 0.85 | Ulnar Neuropathy Elbow | segment;ulnar;across;elbow;velocity;slow;localized;elbows;neuropathies; |
| 5 | 284 | 24 | 0.76 | CTS Moderate | comes;latencies;prolonged;from;median;tunnels;moderate;support;conclusion; |
| 6 | 278 | 19 | 0.79 | LDPN **Symmetric** Sensory Polyneuropathy | dependent;length;symmetric;reduces;extremity;sensorimotor;lower;conclusion;cmaps; |
| 7 | 270 | 9 | 0.72 | Peroneal Neuropathy | ta;deep;peroneal;longus;fibula;peroneus;common;knee;head; |
| 8 | 252 | 15 | 0.62 | Chronic Radiculopathy Cervical | chornic;radiculopathy;palmar;cervical;chronic;non;highly;palsy;localizable; |
| 9 | 252 | 7 | 0.61 | Brachial Plexopathy | renervation;brachial;plexus;axillary;good;plexopathy;complite;cord;antebrachial; |
| 10 | 229 | 10 | 0.62 | DP with axonal loss severe | compatible;gbs;aidp;cidp;hereditary;demyelinating;hnpp;starting;sensorimotor; |
| 11 | 222 | 15 | 0.36 | Absent LFCN/Meralgia | lfcn;lfc;paresthetica;meralgia;obesity;cutaneous;technical;age;legs; |
| 12 | 215 | 25 | 0.43 | Radiculopathy (L5, L4, L3, S1, S2) | share;myotome;finding;reinnervation;background;inactive;that;myotomes;chronic; |
| 13 | 215 | 12 | 0.37 | Motor Neuron Disease | sclerosis;multifocal;radiologic;later;regions;amyotrophic;meet;body;includes; |
| 14 | 205 | 20 | 0.68 | Myasthenia/NMJ | jitter;myasthenia;usec;gravis;nmj;pathologic;toxin;botulin;neuro; |
| 15 | 204 | 3 | 0.87 | Ulnar Neuropathy Elbow | locazlied;localized;elbow;segment;palmar;across;ulnar;velocity;carpal; |
| 16 | 198 | 1 | 0.70 | CTS | peak;syndrome;syndromes;also;pollicis;worse;wrists;slowed;small; |
| 17 | 193 | 18 | 0.39 | Myopathy/ Myotonia | myotonia;myopathy;voluntary;activity;myotonic;spontaneous;irritable;myopathic;critical; |
| 18 | 183 | 3 | 0.70 | Multiple Entrapment Neuropathies | tunnels;localized;palmar;neuropathies;two;comes;elbow;ulnar;bilateral; |
| 19 | 176 | 2 | 0.80 | CTS-Mild | palmar;stimulation;seen;only;very;botulin;pathologic;locazlied;neuromuscular; |
| 20 | 157 | 18 | 0.55 | Irritable/ Non-Irritable Myopathy | irritable;myositis;myopathy;illness;myotonia;myopathic;diffuse;critical;disuse; |
| 21 | 154 | 17 | 0.43 | Comparison to Previous Study | comparison;previous;improvement;worsening;gbs;residual;now;cidp;significant; |
| 22 | 154 | 17 | 0.30 | Comparison to Previous Study | comparison;previous;significant;without;worsening;residual;palmar;improvement;today; |
| 23 | 139 | 7 | 0.38 | Radial Neuropathy Spiral Groove | edc;spiral;groove;renervation;fcu;de;nervation;complite;below; |
| 24 | 102 | 22 | 0.60 | Chronic Radiculopathy Lumbar | cutaneous;chornic;paresthetica;meralgia;stenosis;criterion;radiculopathy;lumbar;injection; |
